# A method for estimating width bands of variables in economics under uncertainty conditions

**DOI:** 10.1016/j.mex.2020.101184

**Published:** 2020-12-14

**Authors:** Reza Ashraf Ganjoei, Hossein Akbarifard, Mashaallah Mashinchi, Sayyed Abdol Majid Jalaee Esfandabadi

**Affiliations:** a Shahid Bahonar University of Kerman, Kerman, Iran; b Faculty of Management and Economics, Shahid Bahonar University of Kerman, Kerman, Iran; c Department of Statistics, Faculty of Mathematics and Computer, Shahid Bahonar University of Kerman, Kerman, Iran; d Department of Economics, Faculty of Management and Economics, Shahid Bahonar University of Kerman, Kerman, Iran

**Keywords:** FLSTAR model, Bounds, Gini coefficient

## Abstract

This study develops a method to estimate the width bands of variables in economics by fuzzy logic. One of its important features is flexibility in the conditions of economic uncertainty, which can be used to model the uncertainty of external and internal factors on economic variables. In this study, for example, the effect of uncertainty of external factors on the Gini coefficient (income distribution) is investigated. For this purpose, we use the fuzzy logistic smooth transition autoregressive (FLSTAR) model and the Gini coefficient is estimated in three bounds (high, middle and low). The result of this estimation suggest that by appropriate policy making the Gini coefficient can be decreased to the lower bound. Another results of this study is that the authorities should prevent the increase of the Gini coefficient in the middle and upper bands with proper planning for the future. In brief,

• This study introduces a novel method for estimating high, low and middle bounds of economic variables under uncertainty conditions.
• One practical results of this method is to compare high, medium, and low bands of the variables with their current trends, which is a benchmark for policymaking and evaluating the effectiveness of government's policies.
• Programs designed with this method are fast and have low cost

## Graphical abstract

**Figure:**
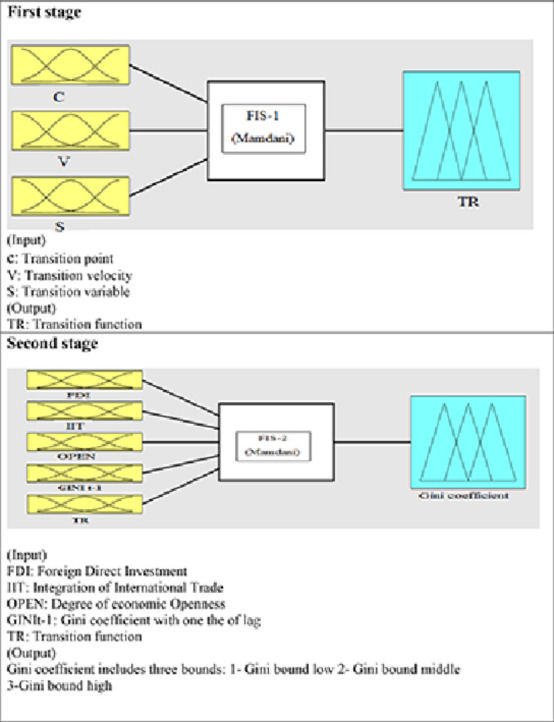

Specifications table

**Table:** 

Subject Area:	Economics and Finance
More specific subject area:	Fuzzy Logic; Modeling uncertainty in economics
Method name:	Fuzzy logistic smooth transition autoregressive (FLSTAR)
Name and reference of original method:	[1] Zadeh, Lofti A. Fuzzy sets, Information and Control 8.3 (1965): 338–353.‏ [2] Terasvirta, T., 1994. Specification, estimation, and evaluation of smooth transition auto- regressive models. Journal of the American Statistical Association 89, 208–218. [3] Tsay, Ruey S. Testing and modeling threshold autoregressive processes. Journal of the American Statistical Association 84.405 (1989): 231–240
Resource availability:	www.cbi.ir www.mathwork.com

## Data description

**Fig. 1:**
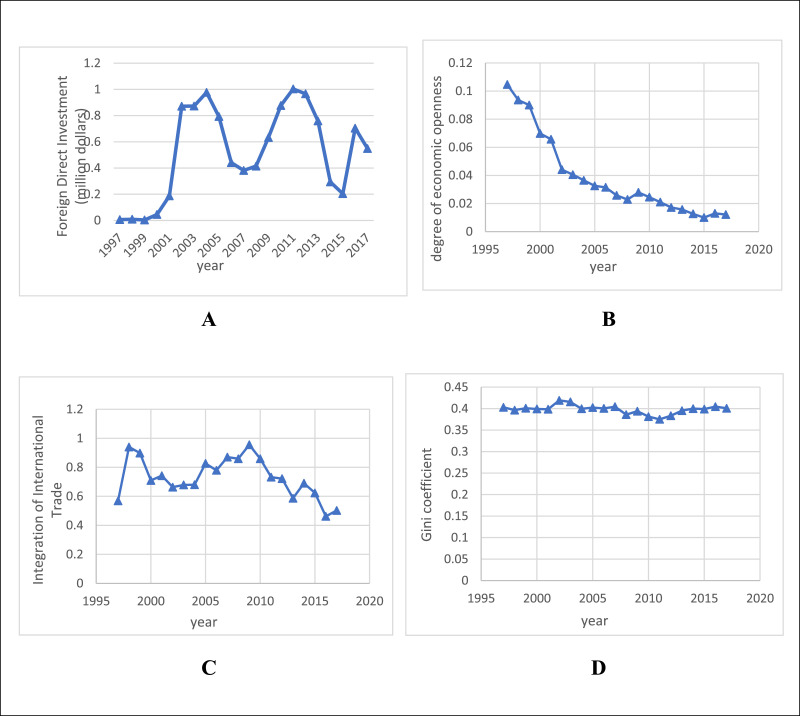
A: Foreign direct investment (FDI), B: Degree of economic openness (OPEN), C: Integration of international trade (IIT), D: Gini coefficient.

In this research, using the annual data during 1997- 2017 presented by the Central Bank of Iran (CBI; https://tsd.cbi.ir/DisplayEn/Content.aspx in [1]), the effect of foreign trade [2] as the degree of economic openness (OPEN), foreign direct investment (FDI), and integration of international trade (IIT), which represents the size of export and import of a country with other countries, is investigated via the Gini coefficient in Iran. Fig. 1-A, B and C shows FDI, OPEN and IIT, in which OPEN is decreasing over time and FDI has an unstable behavior. IIT fluctuates over time, but has declined in recent years. Fig. 1-D shows the trend of the Gini coefficient over time. The trend of this variable is stable over time.

## Method details

### Autoregressive models

One view on autoregressive models is that they provide good first-order approximations to the dynamics of the data-generating process. Statistical modeling of time series [3] is one of the oldest and most successful tools for predicting the future values of a time series as a combination of past values. Box and Jenkins [4] stated the future values of a time series as a linear combination of its past values in the form of an autoregressive (AR) model based on *p* ≥ 1 lagged values of y_t_, where p is the maximum order of the lag, as defined in (1):

(1) $$y_{t}=b^{\prime }x_{t}+\varepsilon_{t}=b_{0}+b_{1}y_{t-1}+\ldots +b_{p}y_{t-p}+\varepsilon_{t},\;t=1,2,\ldots ,n$$

where $b^{\prime }$ is vector of parameters, The symbol ´on the vector means its transpose vector **X***_t_* $={\left(\;1,y_{t-1,\ldots \ldots \ldots ,\;}y_{t-p}\right)}^{{}^{\prime }}\;\text{and}\;\varepsilon_{t}∼N\left(0,\sigma^{2}\right)$ is usually known as white noise (or a random signal). For this model we write, y_t_ ~AR)p), and $\left\{y_{t}\right\}$ generated from this model is called the AR)p) process. Model (1) indicates the current status of $y_{t}$ through the past values of $y_{t-1,\ldots \ldots \ldots ,\;}y_{t-p}$ in terms of a linear regression. This explicitly specifies the relationship between its current and past values. Box and Jenkins' [3,4] method covers a wide range of scientific fields such as biology, astronomy, and econometrics. Tong [5] proposed a nonlinear model called the threshold autoregressive model (TAR) which is divided into several models based on space-state idea and each is modeled by the autoregressive model. A TAR model with k (k ≥2) regimes is defined as (2):

(2) $$y_{t}=\sum_{i=1}^{k}{b^{\prime }}_{i}X_{t}\;I\left(S_{t}\in A_{i}\right)+\varepsilon_{t}=\sum_{i=1}^{k}\left\{b_{i,0}+b_{i,1}y_{t-1}+b_{i,p}y_{t-p}\right\}I\left(S_{t}\in A_{i}\right)+\varepsilon_{t},$$

where $S_{t}$ is threshold variable, I is indicator function with values 0 and 1, $b_{i}^{{}^{\prime }}\;$ is a vector of parameters, and $\left\{A_{i}\right\}$ are the partition of the real line R= (-∞, ∞), so that:

(3) $$\cup_{i=1}^{k}A_{i}=\left(-\infty ,\infty \right)\;and\;A_{i}\cap A_{j}=\emptyset ,\forall_{i}\neq j,$$

Here for each set $A_{i}=\left(r_{i-1},r_{i}\right)$ of the partition R, the model is expressed in an autoregressive form. This the partition is characterized by the threshold variable $S_{t}$ and the threshold limits *r_i,_* where$\;\;-\infty =r_{0}<r_{1}<\ldots <r_{k}=\infty$ [3].

## Smooth transition autoregressive model

One of the key features of threshold autoregressive models is the discontinuous correlation of the autoregressive model. An alternative model called smooth transition autoregressive (STAR) model was proposed by Teräsvirta [3,6]. This model with *k* numbers of regimes is defined in (4),

(4) $$y_{t}=\sum_{i=1}^{k}{b^{\prime }}_{i}X_{t}\;f_{i}\left(S_{t};\emptyset_{i}\right)+\varepsilon_{t\;},$$

where$\;b_{i}$ is a vector of parameters, $f_{i}\left(S_{t};\emptyset_{i}\right)$ is transition function,$\;\emptyset_{i}$ consists of two variables γ and c, where γ represents the transition velocity between two bounds, and c is the transition point and $\{\varepsilon_{t}\}\;$*~ N*$(0,\sigma^{2})\;$usually known as white noise (equivalent to a random signal with a flat power spectral density)

The STAR model in (4) can be re-written as in (5),

(5) $$y_{t}=\sum_{i=1}^{k}{b^{\prime }}_{i}\;X_{t}F\left(S_{t};\gamma_{i},c_{i}\right)+\varepsilon_{t},$$

where, $\gamma_{i}$ represents the transition velocity and $c_{i}$ is the transition point [3].

## Fuzzy logic methodology

Fuzzy sets were introduced by L. A. Zadeh [7]. After introducing this notion, the fuzzy data were used for modeling uncertain information in the databases. Fuzzy logic involves a wide range of theories and techniques that are generally based on four concepts: fuzzy sets, verbal variables, membership function, and fuzzy if-then rules [8,9]. The fuzzy logic consists of three stages as fuzzification, fuzzy process (fuzzy inference) and defuzzification [10]. In this Section, we will review the application of fuzzy logic in prediction and modeling.

### Fuzzy-based models

Fuzzy systems are knowledge or rule-based systems [3,11]. The heart of a fuzzy system is a knowledge based that is formed by fuzzy if-then rules. A fuzzy if-then rule is a conditional expression which is specified by continuous membership functions [3]. The fuzzy inference system (IFS) produces outputs based on inputs and by using fuzzy rules [3]. While dealing with time series problems, the Takagi-Sugeno-Kang (TSK) model is preferred to the other types. The TSK type fuzzy rule [3] is as in (6),

(6) $$\begin{array}{c} IF\;x_{1}\;is\;A_{1}\;AND\;x_{2}\;is\;A_{2}\;AND\ldots AND\;x_{p}is\;A_{p}\\ THEN\;y=b^{\prime }x_{t}=b_{0}+b_{1}x_{1}+b_{2}x_{2}+\ldots +b_{p}x_{p,} \end{array}$$

where$\;x_{j}$ is input variable and *A_j_* is a fuzzy set. Given the fuzzy argumentation mechanism for the TSK rules, the firing strength of the i^th^ rule is obtained as the t-norm (usually, multiplication operator) of the membership values of the premise part terms of the linguistic variables as in (7),

(7) $$\omega \left(x\right)=\prod_{j=1}^{p}\mu_{Aj}\left(x_{j}\right),\text{with}\;x\;=\left(x_{1},\ldots ,x_{p}\right)$$

The membership function $\mu_{\text{Aj}\;}\;$can be selected from a wide range of functions [3]. One of the most common ones is the Gaussian bell shaped as presented in (8):

(8) $$\mu_{A}\left(x\right)=\frac{1}{\sigma \sqrt{2\pi }}e^{-\frac{1}{2}{\left(\left(x-\mu \right)/\sigma \right)}^{2}},$$

However, it can also be a logistic function [3] as in (9):

(9) $$\mu_{A}\left(x\right)=\frac{1}{1+exp\left(\frac{c-x}{\sigma }\right)}$$

The consequent is calculated as the average weight or total output weight of the rules. In the case of the total weight, the output is stated as in (10):

(10) $$y_{t}=G\left(x_{t};\Psi \right)=\sum_{i=1}^{R}{b^{\prime }}_{i}X_{t}\omega_{i}\left(x_{t}\right),$$

where G is the general nonlinear function with parameters ᴪ and *R* is the number of fuzzy rules in the system [3]. When a univariate time series $\{y_{t}\}\;$ is used for modeling or predicting, the TSK type fuzzy-based rules are expressed as in rule (11); all the variables $y_{t-i}\;$ are lagged values of the time series $\left\{y_{t}\right\}$.

(11) $$\begin{array}{c} IF\;y_{t-1}\;is\;A_{1}\;AND\;y_{t-2}\;is\;A_{2}AND\ldots AND\;y_{t-p}\;is\;A_{p}\\ THEN\;y_{t}=b_{0}+b_{1}y_{t-1}+b_{2}y_{t-2}+\ldots +b_{p}y_{t-p}. \end{array}$$

## THE modelling CYCLE

In Sections 4.1, we summarize the basics of nonlinear models and fuzzy rules. The main focus here is the expression of the steps of the modelling cycle for calculating the upper, middle and lower bands of the Gini coefficient performed in Windows 10, 64-bit and MATLAB R2019a, which are as follows [12]:

### Specifications of the LSTAR model

The logistic smooth transition autoregressive (LSTAR) model regime switches are associated with small and large values of the transition variable $S_{t}$ relative to *c*. In certain applications it may be more appropriate to specify the transition function such that the regimes are associated with small and large absolute values of $S_{t}$ (again relative to *c*). The transition variable $S_{t}$ is a stochastic variable that is selected from among the independent variables or dependent variable [3] .In the LSTAR model, the transition function $F(S_{t};\gamma_{i},c_{i})\;$in Equation (12)isdefinedas:

(12) $$F_{i}\left(S_{t};\gamma_{i},c_{i}\right)=\left\{ \begin{array}{cc} 1-f_{l}\left(S_{t};\gamma_{i},c_{i}\right) & if\;i=1,\\ f_{l}\left(S_{t};\gamma_{i},c_{i}\right)-f_{l}\left(S_{t};\gamma_{i+1},c_{i+1}\right) & if\;1<i<k,\\ f_{l}\left(S_{t};\gamma_{i},c_{i}\right) & if\;i=k, \end{array}\right.$$

where the transition function is specified as in (13), Where $f_{l}\left(S_{t};\gamma_{i},c_{i}\right)={\left(1+exp(\gamma_{i}\left(S_{t}-c_{i}\right))\right)}^{-1}$. The LSTAR model can be consequently rewritten as

(13) $$y_{t}=\sum_{i=1}^{k}{b^{\prime }}_{i}x_{t}F\left(S_{t};\gamma_{i},c_{i}\right)+\varepsilon_{t},$$

where the transition function is specified as in (12). Parameters $c_{i}$ in (13)can be interpreted as the threshold between two regimes, in the sense that the logistic function changes monotonically from 0 to1as $S_{t}$increases and $F(S_{t};\gamma_{i},c_{i})\;$= 0.5. Parameter$\;\gamma_{i}$ determines the smoothness of the transition from one regime to another. As $\;\gamma_{i}$ becomes very large, the logistic function approaches the indicator function I (•); hence the change of $F(S_{t};\gamma_{i},c_{i})$ from 0 to 1 becomes instantaneous at $S_{t}$ → *c*. The LSTAR threshold autoregressive (TAR) models as a special case. Furthermore, when γ → 0 the LSTAR model reduces to a linear AR model [3].In this section, we will extend the methods used in previous studies [13,14]. The methodological proposal is to use a fuzzy rule-based system (FRBS) on a smooth transition autoregressive (STAR) model, the transition function of which is specified as the first-order logistic function [15].

$$G\left(S_{t};v,c\right)={\left(1+exp\left\{-v\left(S_{t}-c\right)\right\}\right)}^{-1},v>0\;\left(\text{identifying}\;\text{restriction}\right),$$

where:

• $S_{t}$ is a transition variable, chosen in this manuscript as the so-called “degree of economic openness” (OPEN),
• v is a parameter determining the smoothness of the change in the value of the logistic function, and
• *c* is a threshold parameter.

If the transition function is specified as

$$G\left(S_{t};v,c\right)=1-e^{-v{\left(S_{t}-c\right)}^{2}},v>0,$$

then the resulting model is referred to as exponential STAR (ESTAR) model.

The novelty of this study is to present a method for estimating the width bands of the variables in economics under uncertainty conditions. Modeling the uncertainty of economic variables is very important. Via using the fuzzy rule-based system (FRBS) on LSTAR models with exogenous variables, suitable conditions are provided to investigate the effect of the uncertainty of the variables on the economy. When modeling every problem (for example, modeling the uncertainty of the variables in economics), although there are very complex theoretical foundations, all the complexities of economic system in modeling can be considered by the fuzzy rule-based system (FRBS) on LSTAR models with exogenous variables; this causes an increase in the accuracy of the answers.

### Steps of the modeling cycle for the methodology LSTAR models

The main focus in this section is on modeling the nonlinear behavior of the variables in economics. For this purpose EViews software is used [16]. EViews can be used for general statistical analysis and econometric analyses, such as cross-section, panel data analysis and time series estimation, and forecasting. The EViews software handles nonlinear time series modeling. The input variables include: degree of economic openness (OPEN), foreign direct investment (FDI), and integration of international trade (IIT). In this study, the nonlinear LSTAR model is used to evaluate the behavior of the variables. In order to select the appropriate nonlinear model (LSTAR) in EViews software, we use Taylor's third-degree expansion, Lagrange multiplier (LM)-type tests and testing hypothesis, respectively [17], [18], [19], [20]. The steps of the modeling cycle for the methodology LSTAR models are as follows

**Step 0 - stable variables:** In the time series, the augmented Dickey Fuller test is the test trying to analyze whether the variables have unit roots or not. In 1974, Granger and Newbold found that there could have been a spurious regression problem if the variables are not stable.

ADF unit root test regression equation;

$$\Delta Y_{t}=\left(\rho -1\right)Y_{t-1}+u_{t}=\delta Y_{t-1}+u_{t}$$

where Δ denotes the first difference value of the variable. The model is estimated as a result of the analysis and then the hypothesis $\;H_{0}$: δ= 0 is applied to the variables. Since the variables in the serie are connected to a random variable, the $H_{0}$ hypothesis can be interpreted that the unit root exists, that is, the variables are non-stationary. As the residual values are used in the ADF unit root test, t statistic with a special table is used instead of the standard t statistic value [21].

**Step 1 - Testing linearity:** One of the most important steps in estimating a smooth transfer regression model is to test the linear model against the nonlinear. The main question is that if the model is nonlinear, which process (ESTAR or LSTAR model) does it follow? Therefore, the null hypothesis based on the linear is defined as $H_{0}=0\;,\;v>0\;$ [9,17]. In Eq. (14) when v=0, the LSTAR model reduces to a linear regression [18,20]:

(14) $$G\left(s_{t};v,c\right)={\left(1+exp\left\{-v\left(s_{t}-c\right)\right\}\right)}^{-1},v>0.$$

In this case, the parameters $S_{t}$ and *c* are indefinite. The solution that Luukkonen et al. (1988) [17] and Teräsvirta (1994) [7] have proposed to solve this problem is to replace the transfer function with Taylor's approximation. After replacing the transition function by its third-order Taylor approximation around v = 0, the auxiliary regression model is obtained. Taylor's third-degree expansion is based on the suggestion made by Luukkonen et al. (1988) [17]. Thus, auxiliary regression of Relation (15) is written [22,23] as:

(15) $$Y_{t}=\beta_{0}^{\prime }z_{t}+\sum_{i=1}^{k}\beta_{k}^{\prime }{\bar{z}}_{t}s_{t}^{k}+v_{t},$$

where $z_{t}={\left({W^{\prime }}_{t}\;,{X^{\prime }}_{t}\right)}^{\prime }$ is a vector of explanatory variables, ${W^{\prime }}_{t}={\left(1,y_{t-1},y_{t-2\;,\ldots ,\;\;}y_{t-p}\right)}^{\prime }$and ${X^{\prime }}_{t}={\left(x_{1t,\ldots ,}x_{kt}\right)}^{\prime }$ are vectors of exogenous variables, $S_{t}$ is the transfer variable, $\beta_{0}^{\prime }\;$ linear coefficients of the auxiliary model, $\beta_{k}^{\prime }\;$non-linear coefficients of the auxiliary model, $z_{t}=\left(1,\bar{z_{t}}\right)$, where $\bar{z_{t}}$ is a (m × 1) vector and $v_{t}$ ≈ *iid* (0, $\sigma^{2}$), where the Lagrange multiplier (LM)-type tests is defined as:

$$LM_{W}=\frac{T\left(\text{SS}R_{0}-\text{SS}R_{1}\right)}{\text{SS}R_{0}},$$

where, $\text{SS}R_{0}$ is the sum of residuals squared, SSR1 is the sum of squared residuals and, T is time period. (LM)-type test can carried out in stages [3]:

(1) Regress y*_t_* on **x***_t_* and compute the residual sum of squares $SSR_{0}=\sum_{t=1}^{T}{\hat{\sigma }}_{t}^{2}$ where σ is the variance of *ɛ*
(2) Regress ${\hat{\sigma }}_{t}$ on **x***_t_* and on the m (degrees of freedom) nonlinear regressors of (16). Compute the residual sum of squares$\;SSR_{1}=\sum_{t=1}^{T}{\hat{\tau }}_{t}^{2}$

where ${\hat{\tau }}_{t}^{2}$ is contains all the nonlinear regressors in (16),

(16) $$y_{t}=\pi^{\prime }x_{t}+\sum_{i=1}^{P}\sum_{j=1}^{P}\beta_{ij}x_{i}x_{j}+\sum_{i=1}^{P}\sum_{j=i}^{P}\sum_{k=j}^{P}\beta_{ijk}x_{i}x_{j}x_{k}+\varepsilon_{t}^{*}$$

where $\beta_{ij\;}$,$\;\beta_{ijk}\;$ are coefficients,$\;\varepsilon^{*}=b_{1}x_{t}R\left(x_{t};\gamma \;,\;c\right)$,which means that $\varepsilon^{*}=\;$*ɛ* under the null hypothesis. In this case, the null hypothesis is related to the linear relationship written as Eq. (13).

$$H_{0}=\beta_{1}^{\prime }=\beta_{2}^{\prime }=\beta_{3}^{\prime }=0$$

We use Akaike, Bayesian or Schwarz, and Hannan-Quinn criteria (henceforth AIC, BIC, and HQ) for determine the optimal lag. According to this criterion, the optimal lag for the variables is one. Table 1 presents the results of criteria AIC, BIC, and HQ.

**Table 1:** Optimal lag determination.

The number of lag	Criteria		
	AIC	BIC	HQ
0	−3.615	−4.019	−3.527
1	−4.718*	−4.834*	−5.230
2	−3.507	−3.516	−4.540
3	−4.315	−3.314	−3.517

*Note:* Computing Akaike, Bayesian (AIC) or Schwarz (BIC), and Hannan-Quinn (HQ) criteria for the optimal lag.

We now write the functions required for Taylor's first to third approximations that we need in this study. In Eqs. (17) to (28), $\beta_{it}$, *i* = 0, 1, 2, 3 and *t* = 1, 2 , … , 5 are real coefficients to be estimated, $Gini_{t}\;\text{is}\;\text{Gini}\;\text{coefficient},\;$ FDI is foreign direct investment, IIT is integration of international trade and OPEN is the degree of economic openness and $Gini_{t-1}$ is Gini coefficient of the previous period (estimation results are in appendix). For the foreign direct investment variable, the first to third Taylor's approximations are as (17), (18), (19),

(17) $$\begin{array}{ccc} Gini_{t} & = & \beta_{01}+\beta_{02}Gini_{t-1}+\beta_{03}FDI_{t-1}+\beta_{04}OPEN_{t-1}+\beta_{05}IIT_{t-1}+\beta_{11}\left(FDI_{t-1}*Gini_{t-1}\right)+\beta_{12}\left(FDI_{t-1}^{2}\right)\\ & & +\;\beta_{13}\left(FDI_{t-1}*OPEN_{t-1}\right)+\beta_{14}\left(FDI_{t-1}*IIT_{t-1}\right)+\varepsilon_{t,} \end{array}$$

(18) $$\begin{array}{ccc} Gini_{t} & = & \beta_{01}+\beta_{02}Gini_{t-1}+\beta_{03}FDI_{t-1}+\beta_{04}OPEN_{t-1}+\beta_{05}IIT_{t-1}+\beta_{11}\left(FDI_{t-1}*Gini_{t-1}\right)+\beta_{12}\left(FDI_{t-1}^{2}\right)\\ & & +\;\beta_{13}\left(FDI_{t-1}*OPEN_{t-1}\right)+\beta_{14}\left(FDI_{t-1}*IIT_{t-1}\right)+\beta_{21}\left(Gini_{t-1}*FDI_{t-1}^{2}\right)+\beta_{22}\left(FDI_{t-1}^{3}\right)\\ & & +\;\beta_{23}\left(OPEN_{t-1}*FDI_{t-1}^{2}\right)+\beta_{24}\left(IIT_{t-1}*FDI_{t-1}^{2}\right)+\varepsilon_{t,} \end{array}$$

(19) $$\begin{array}{ccc} Gini_{t} & = & \beta_{01}+\beta_{02}Gini_{t-1}+\beta_{03}FDI_{t-1}+\beta_{04}OPEN_{t-1}+\beta_{05}IIT_{t-1}+\beta_{11}\left(FDI_{t-1}*Gini_{t-1}\right)+\beta_{12}\left(FDI_{t-1}^{2}\right)\\ & & +\;\beta_{13}\left(FDI_{t-1}*OPEN_{t-1}\right)+\beta_{14}\left(FDI_{t-1}*IIT_{t-1}\right)+\beta_{21}\left(Gini_{t-1}*FDI_{t-1}^{2}\right)+\beta_{22}\left(FDI_{t-1}^{3}\right)\\ & & +\;\beta_{23}\left(OPEN_{t-1}*FDI_{t-1}^{2}\right)+\beta_{24}\left(IIT_{t-1}*FDI_{t-1}^{2}\right)+\beta_{31}\left(Gini_{t-1}*FDI_{t-1}^{3}\right)+\beta_{32}\left(FDI_{t-1}^{4}\right)\\ & & +\;\beta_{33}\left(FDI_{t-1}^{3}*OPEN_{t-1}\right)+\beta_{34}\left(FDI_{t-1}^{3}*IIT_{t-1}\right)+\varepsilon_{t.} \end{array}$$

For the degree of economic openness, Taylor's first to third approximations are as (20), (21), (22),

(20) $$\begin{array}{ccc} Gini_{t} & = & \beta_{01}+\beta_{02}Gini_{t-1}+\beta_{03}FDI_{t-1}+\beta_{04}OPEN_{t-1}+\beta_{05}IIT_{t-1}+\beta_{11}\left(OPEN_{t-1}*Gini_{t-1}\right)\\ & & +\;\beta_{12}\left(OPEN_{t-1}*FDI_{t-1}\right)+\beta_{13}\left(OPEN_{t-1}^{2}\right)+\beta_{14}\left(OPEN_{t-1}*IIT_{t-1}\right)+\varepsilon_{t,} \end{array}$$

(21) $$\begin{array}{ccc} Gini_{t} & = & \beta_{01}+\beta_{02}Gini_{t-1}+\beta_{03}FDI_{t-1}+\beta_{04}OPEN_{t-1}+\beta_{05}IIT_{t-1}\\ & & +\;\beta_{11}\left(OPEN_{t-1}*Gini_{t-1}\right)\beta_{12}\left(OPEN_{t-1}*FDI_{t-1}\right)\\ & & +\;\beta_{13}\left(OPEN_{t-1}^{2}\right)+\beta_{14}\left(OPEN_{t-1}*IIT_{t-1}\right)\\ & & +\;\beta_{21}\left(Gini_{t-1}*OPEN_{t-1}^{2}\right)+\beta_{22}\left(FDI_{t-1}*OPEN_{t-1}^{2}\right)\\ & & +\;\beta_{23}\left(OPEN_{t-1}^{3}\right)+\beta_{24}\left(IIT_{t-1}*OPEN_{t-1}^{2}\right)+\varepsilon_{t,} \end{array}$$

(22) $$\begin{array}{ccc} Gini_{t} & = & \beta_{01}+\beta_{02}Gini_{t-1}+\beta_{03}FDI_{t-1}+\beta_{04}OPEN_{t-1}+\beta_{05}IIT_{t-1}+\beta_{11}\left(OPEN_{t-1}*Gini_{t-1}\right)\\ & & +\;\beta_{12}\left(OPEN_{t-1}*FDI_{t-1}\right)+\beta_{13}\left(OPEN_{t-1}^{2}\right)+\beta_{14}\left(OPEN_{t-1}*IIT_{t-1}\right)+\beta_{21}\left(Gini_{t-1}*OPEN_{t-1}^{2}\right)\\ & & +\;\beta_{22}\left(FDI_{t-1}*OPEN_{t-1}^{2}\right)+\beta_{23}\left(OPEN_{t-1}^{3}\right)+\beta_{24}\left(IIT_{t-1}*OPEN_{t-1}^{2}\right)+\beta_{31}\left(Gini_{t-1}*OPEN_{t-1}^{3}\right)\\ & & +\;\beta_{32}\left(FDI_{t-1}*OPEN_{t-1}^{3}\right)+\;\beta_{33}\left(OPEN_{t-1}^{4}\right)+\beta_{34}\left(IIT_{t-1}*OPEN_{t-1}^{3}\right)+\varepsilon_{t.} \end{array}$$

for the integration of international trade, Taylor's first to third approximations are as (23), (24), (25); and

(23) $$\begin{array}{ccc} Gini_{t} & = & \beta_{01}+\beta_{02}Gini_{t-1}+\beta_{03}FDI_{t-1}+\beta_{04}OPEN_{t-1}+\beta_{05}IIT_{t-1}+\beta_{11}\left(IIT_{t-1}*Gini_{t-1}\right)\\ & & +\;\beta_{12}\left(IIT_{t-1}*FDI_{t-1}\right)+\beta_{13}\left(IIT_{t-1}*OPEN_{t-1}\right)+\beta_{14}\left(IIT_{t-1}^{2}\right)+\varepsilon_{t,} \end{array}$$

(24) $$\begin{array}{ccc} Gini_{t} & = & \beta_{01}+\beta_{02}Gini_{t-1}+\beta_{03}FDI_{t-1}+\beta_{04}OPEN_{t-1}+\beta_{05}IIT_{t-1}+\beta_{11}\left(IIT_{t-1}*Gini_{t-1}\right)\\ & & +\;\beta_{12}\left(IIT_{t-1}*FDI_{t-1}\right)+\beta_{13}\left(IIT_{t-1}*OPEN_{t-1}\right)+\beta_{14}\left(IIT_{t-1}^{2}\right)+\beta_{21}\left(Gini_{t-1}*IIT_{t-1}^{2}\right)\\ & & +\;\beta_{22}\left(FDI_{t-1}*IIT_{t-1}^{2}\right)+\beta_{23}\left(OPEN_{t-1}*IIT_{t-1}^{2}\right)+\beta_{24}\left(IIT_{t-1}^{3}\right)+\varepsilon_{t,} \end{array}$$

(25) $$\begin{array}{ccc} Gini_{t} & = & \beta_{01}+\beta_{02}Gini_{t-1}+\beta_{03}FDI_{t-1}+\beta_{04}OPEN_{t-1}+\beta_{05}IIT_{t-1}+\beta_{11}\left(IIT_{t-1}*Gini_{t-1}\right)\\ & & +\;\beta_{12}\left(IIT_{t-1}*FDI_{t-1}\right)+\beta_{13}\left(IIT_{t-1}*OPEN_{t-1}\right)+\beta_{14}\left(IIT_{t-1}^{2}\right)+\beta_{21}\left(Gini_{t-1}*IIT_{t-1}^{2}\right)\\ & & +\;\beta_{22}\left(FDI_{t-1}*IIT_{t-1}^{2}\right)+\beta_{23}\left(OPEN_{t-1}*IIT_{t-1}^{2}\right)+\beta_{24}\left(IIT_{t-1}^{3}\right)+\beta_{31}\left(Gini_{t-1}*IIT_{t-1}^{3}\right)\\ & & +\;\beta_{32}\left(FDI_{t-1}*IIT_{t-1}^{3}\right)+\beta_{33}\left(OPEN_{t-1}*IIT_{t-1}^{3}\right)+\beta_{34}\left(IIT_{t-1}^{4}\right)+\varepsilon_{t.} \end{array}$$

for Gini coefficient with one the of lag, Taylor's first to third approximations are as (26), (27), (28).

(26) $$\begin{array}{ccc} \text{Gin}i_{t} & = & \beta_{01}+\beta_{02}\text{Gin}i_{t-1}+\beta_{03}FDI_{t-1}+\beta_{04}OPEN_{t-1}+\beta_{05}IIT_{t-1}+\beta_{11}\left({\text{Gini}}_{t-1}^{2}\right)+\beta_{12}\left(\text{FD}I_{t-1}*\text{Gin}i_{t-1}\right)\\ & & +\;\beta_{13}\left(OPEN_{t-1}*\text{Gin}i_{t-1}\right)+\beta_{14}\left(\text{II}T_{t-1}*\text{Gin}i_{t-1}\right)+\varepsilon_{t,} \end{array}$$

(27) $$\begin{array}{ccc} \text{Gin}i_{t} & = & \beta_{01}+\beta_{02}\text{Gin}i_{t-1}+\beta_{03}FDI_{t-1}+\beta_{04}OPEN_{t-1}+\beta_{05}IIT_{t-1}+\beta_{11}\left({\text{Gini}}_{t-1}^{2}\right)+\beta_{12}\left(\text{FD}I_{t-1}*\text{Gin}i_{t-1}\right)\\ & & +\;\beta_{13}\left(OPEN_{t-1}*\text{Gin}i_{t-1}\right)+\beta_{14}\left(\text{II}T_{t-1}*\text{Gin}i_{t-1}\right)+\beta_{21}\left({\text{Gini}}_{t-1}^{3}\right)+\beta_{22}\left(FDI_{t-1}*{\text{Gini}}_{t-1}^{2}\right)\\ & & +\;\beta_{23}\left(OPEN_{t-1}*{\text{Gini}}_{t-1}^{2}\right)+\beta_{24}\left(\text{II}T_{t-1}*{\text{Gini}}_{t-1}^{2}\right)+\varepsilon_{t,} \end{array}$$

(28) $$\begin{array}{ccc} \text{Gin}i_{t} & = & \beta_{01}+\beta_{02}\text{Gin}i_{t-1}+\beta_{03}FDI_{t-1}+\beta_{04}OPEN_{t-1}+\beta_{05}IIT_{t-1}+\beta_{11}\left({\text{Gini}}_{t-1}^{2}\right)\\ & & +\;\beta_{12}\left(\text{FD}I_{t-1}*\text{Gin}i_{t-1}\right)+\beta_{13}\left(OPEN_{t-1}*\text{Gin}i_{t-1}\right)+\beta_{14}\left(\text{II}T_{t-1}*\text{Gin}i_{t-1}\right)+\beta_{21}\left({\text{Gini}}_{t-1}^{3}\right)\\ & & +\;\beta_{22}\left(FDI_{t-1}*{\text{Gini}}_{t-1}^{2}\right)+\;\beta_{23}\left(OPEN_{t-1}*{\text{Gini}}_{t-1}^{2}\right)+\beta_{24}\left(\text{II}T_{t-1}*{\text{Gini}}_{t-1}^{2}\right)+\beta_{31}\left({\text{Gini}}_{t-1}^{4}\right)\\ & & +\;\beta_{32}\left(FDI_{t-1}*{\text{Gini}}_{t-1}^{3}\right)+\beta_{33}\left(OPEN_{t-1}*{\text{Gini}}_{t-1}^{3}\right)+\beta_{34}\left(\text{II}T_{t-1}*{\text{Gini}}_{t-1}^{3}\right)+\varepsilon_{t.} \end{array}$$

**Step 2 - Choice of the transition variable:** In order to select the transfer variable, according to Taylor's first to third approximations, $\;LM_{W}$ statistic [20] is calculated for each of the independent variables. The variable that has the highest $\;LM_{W}$ test statistic among the other variables is selected as the transfer variable [19]. For further reading see [19,24].

**Step 3 - Choice of the transition function:** If the model is non-linear, the appropriate form must be selected for the transfer function. For this purpose, the following hypotheses are tested in order:

$$\begin{array}{c} H_{1}:{\beta^{\prime }}_{3}=0\\ H_{2}:\;{\beta^{\prime }}_{2}=0|{\beta^{\prime }}_{3}=0\\ H_{3}:\;{\beta^{\prime }}_{1}=0|{\beta^{\prime }}_{2}=0,{\beta^{\prime }}_{3}=0 \end{array}$$

where **|** the term is conditional.

If the $H_{1}$ hypothesis is rejected, the model will have the LSTAR pattern, and if the $H_{1}$ hypothesis is accepted, the $H_{2}$ hypothesis is tested. If this hypothesis is rejected, the model will have the ESTAR model, otherwise, the hypothesis $H_{3}\;$will be tested. If$\;H_{3}$ is rejected, the model will have the LSTAR model.

**Step 4 -** Transition velocity v and transition point$\;c_{i}$ are determined by using the Newton-Raphson algorithm [21]. Transfer point c and transfer velocity point v, are calculated based on Eq. (28), where $\alpha_{i}$, *i* = 0,1,…,4 and $\beta_{i}$, *i* = 0,1,…,4; also, $\alpha_{i}$ and $\beta_{i}$ are real coefficients of the linear and non-linear parts of the model (29), respectively, the estimates of which are provided in Appendix, Table 13, p.31. $Gini_{t}\;\text{is}\;\text{Gini}\;\text{coefficient},\;$ FDI is foreign direct investment, IIT is integration of international trade and OPEN is the degree of economic openness and $Gini_{t-1}$ is Gini coefficient of the previous period. Thus, we guess the initial approximations of these two points to achieve the convergence among them (estimation results are in Appendix).

(29) $$\begin{array}{ccc} Gini & = & \alpha_{0}+\alpha_{1}Gini_{t-1}+\alpha_{2}FDI_{t-1}+\alpha_{3}IIT_{t-1}+\alpha_{4}OPEN_{t-1}+(\beta_{0}+\beta_{1}Gini_{t-1}+\beta_{2}FDI_{t-1}+\beta_{3}IIT_{t-1}\\ & & +\;\beta_{4}OPEN_{t-1})*{\left(1+exp\left\{-v\left(OPEN_{t-1}-c\right)\right\}\right)}^{-1}+\varepsilon_{t,} \end{array}$$

**Step 5 -** The threshold values of the transition function are calculated based on the following relationship

$$G\left(s_{t};v,c\right)={\left(1+exp\left\{-v\left(s_{t}-c\right)\right\}\right)}^{-1},v>0.$$

The LSTAR model as a regime-switching model can include two thresholds. The amount of thresholds depends on the amount of transition function, so that the value of the transfer function can have values 0 and 1. Parameter c can be interpreted as the thresholds between two regimes. The threshold value in the LSTAR model is related to small and large absolute values of $s_{t}$ (again relative to c). See [20,^24^,25].

### Modeling cycle for the methodology FRBS in the framework of LSTAR models

Today, fuzzy systems are extensively used in various sciences. In this study, MATLAB software [12], is used present the manner of to design a fuzzy model. To enter the fuzzy environment in MATLAB software, we use the fuzzy command. Then the following page appears.

**Fig. 2:**
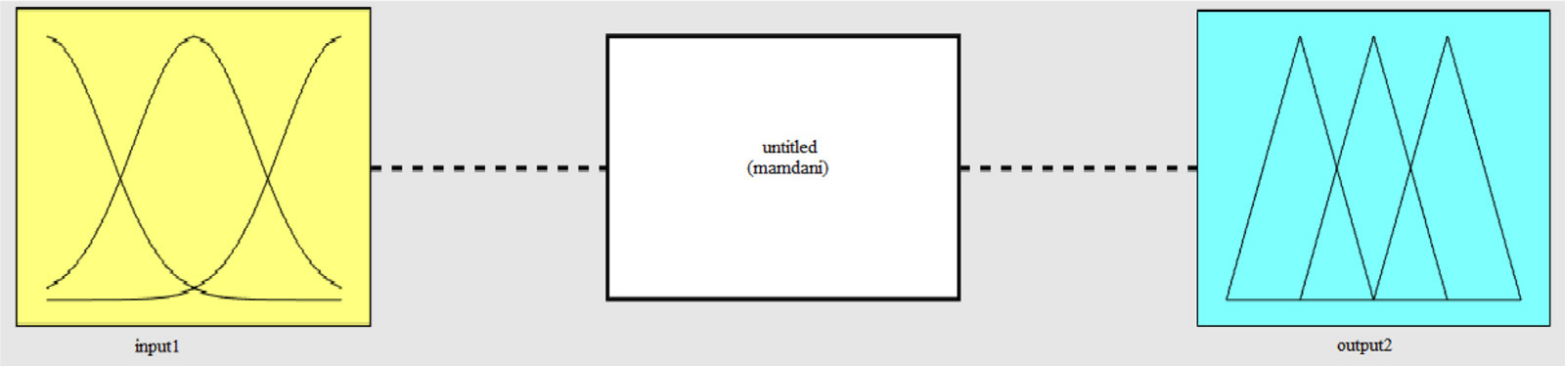
Input membership functions (input1), output membership functions (output2), and inference engine (Mamdani).

On this page, there are input membership functions (input1), output membership functions (output 2), and inference engine (Mamdani) Fig. 2. Fuzzy rules are used in inference engine for creating the acceptable answer**.** In the previous steps (1–5) the LSTAR modeling method was described. In Step 6 the LSTAR estimation method is expressed using fuzzy logic.

**Fig. 3:**
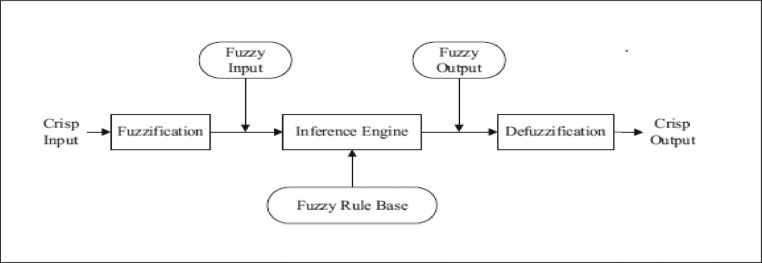
Main components of a fuzzy inference system [10].

**Step 6 -** The modeling cycle for the methodology FRBS in the framework of LSTAR models is as follows. The construction of the fuzzy inference system consists of four parts, which are shown in Fig. 3. For more information.

**Fig. 4:**
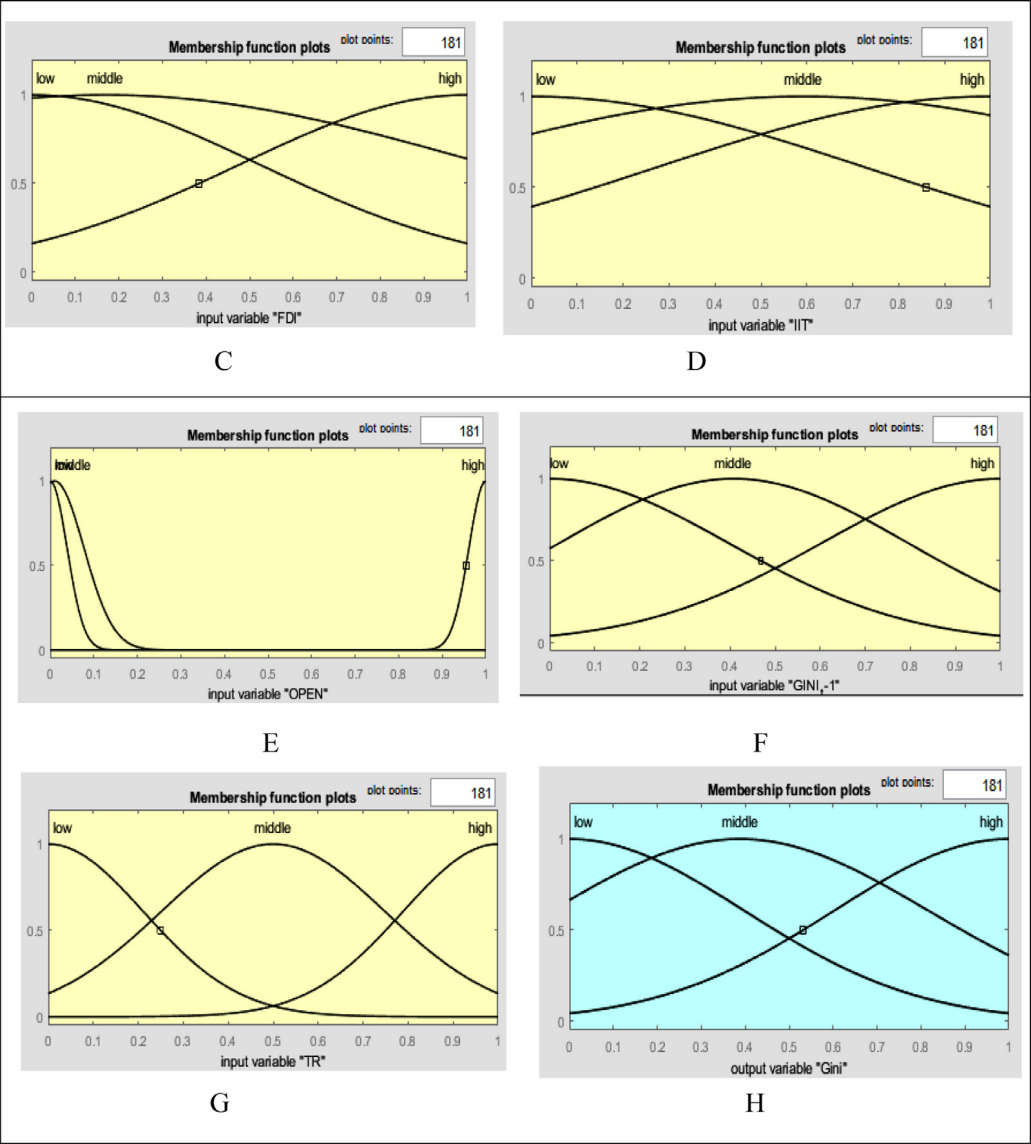
C: membership functions FDI, D: membership functions IIT, E: membership functions OPEN, F: membership functions TR G: Gini coefficient, H: membership functions Gini coefficient with one lag Gini t-1.

**A-Fuzzifier:** The first step in creating a fuzzy system is to define inputs and membership functions. Membership functions should be structured in such a way that they clearly convey the meanings of the relevant linguistic words. In this study, the input and output membership functions is of the Gaussian type written as follows:

$$\mu_{A}\left(x\right)=exp\frac{-{\left(x-c\right)}^{2}}{2\sigma^{2}},\;$$

where x input value σ is the standard deviation and c is the mean inputs. Fig. 4-C, D, E, F and G represents the input membership functions. Fig. 4-H represents the output membership function (as presented in Appendix).

**B-Rule Base:** Fuzzy rule base is a phrase with an if-then structure. The number of rules required depends on the number of indicators and the number of classes in each indicator and is calculated according to the *I* = *m* tob power n, where m is the number of status and n number of input.

Fuzzy rules have two parts: "if part" and "then part"; they are mainly expressed as the following statements:

$$if\;x_{1}\;is\;A_{1}^{r}\;and\;x_{2}\;is\;A_{2}^{r}\;and\;x_{n}\;is\;A_{n}^{r}\;then\;is\;B^{r},$$

**Fig. 5:**
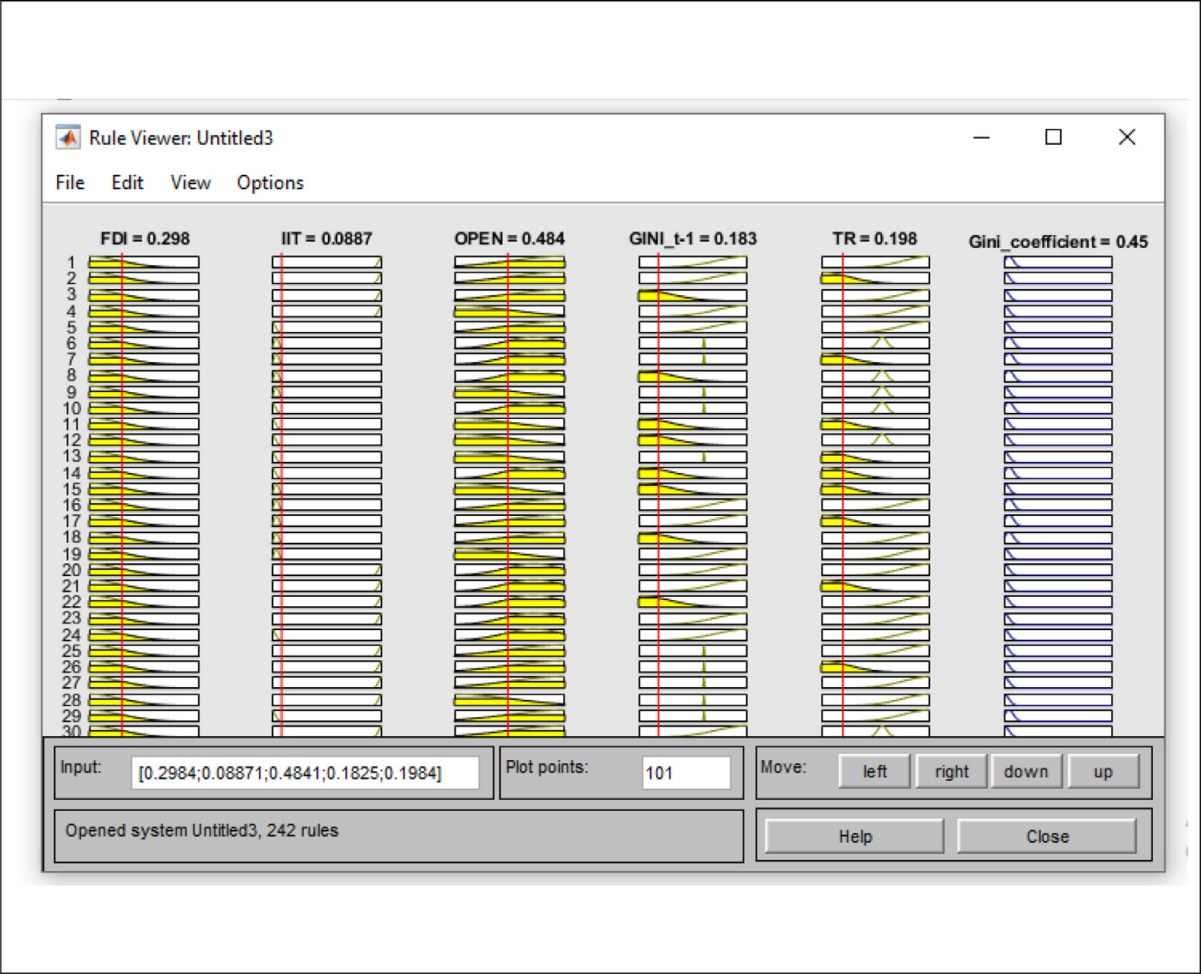
Rule representation of the proposed FIS.

$A_{i}^{r}$ are fuzzy sets such as large, medium, positive large, and *r* represents the *r*^th^ rule. B is the output of the system. In this study, fuzzy rules are written based on Table 2. The representation of the rules in MATLAB software is shown in Fig. 5 (as presented in Appendix).

**Table 2:** Inference rules of FIS.

Rules	IF					THEN	
	FDI	IIT	OPEN	GINIt-1	TR	Gini coefficient	Degree
1	High	High	High	middle	Low	High	1
2	High	High	middle	Low	Low	High	1
3	High	High	Low	middle	Low	High	1
4	middle	High	High	High	Low	Middle	1
5	middle	High	middle	Low	Low	Middle	1
6	middle	High	Low	middle	Low	Middle	1
7	Low	High	High	Low	Low	Middle	1
8	Low	High	middle	High	Low	Middle	1
9	Low	High	Low	Low	Low	Low	1
.	.	.	.	.	.	.	.
.	.	.	.	.	.	.	.
.	.	.	.	.	.	.	
243	Low	Low	Low	middle	middle	Low	1

*Note:* Fuzzy rules based on input variables included: foreign direct investment, degree of economic openness, integration of international trade and output variable Gini coefficient.

**C-Inference engine:** Inference engine is a component of the system that applies logical rules to the knowledge base in order to deduce new information.

**D-Defuzzification:** In this section, the fuzzy output obtained in the previous section is converted into a non-fuzzy number. There are five common ways to do this: centroid, bisector, middle of maximum, smallest of maximum, and largest of maximum.

In this study, the centroid method is used in the defuzzification, and the irregularity is covered by the desired fuzzy number. The centroid method is as follows:

$$output=\;\frac{\int^{x}\;.\mu \left(x\right)dx}{\int^{\mu }\left(x\right)dx},$$

where μ is the fuzzy number membership function of the input.

**Step 7  -**In order to estimate the Gini coefficient of high, middle and low bands in accordance with (30), the inputs are initially analyzed according to the fuzzy-rules base [11] as in (3). The transfer function consists of three parameters of $\tilde{c}$, $\tilde{v}$, and ${\tilde{S}}_{t}$.

(30) $$\begin{array}{ccc} & & {\tilde{Gini}}_{i,t}\left(\;{\tilde{Gini}}_{t-j}\tilde{,FDI},\tilde{OPEN},\tilde{IIT,}{\tilde{S}}_{it},\tilde{c,}\tilde{v}\;\right)\\ & & \;=\sum_{j=1}^{n}\alpha_{j}{\tilde{Gini}}_{t-j}+\sum_{j=1}^{n}\alpha_{j}{\tilde{FDI}}_{t-j}+\sum_{j=1}^{n}\alpha_{j}{\tilde{OPEN}}_{t-j}+\sum_{j=1}^{n}\alpha_{j}{\tilde{IIT}}_{t-j}\\ & & \;+\;\tilde{G}\left({\tilde{S}}_{it},\tilde{v},\tilde{c}\right)*\left\{\;\sum_{j=1}^{n}\alpha_{j}{\tilde{Gini}}_{t-j}+\sum_{j=1}^{n}\alpha_{j}{\tilde{FDI}}_{t-j}+\sum_{j=1}^{n}\alpha_{j}{\tilde{OPEN}}_{t-j}+\sum_{j=1}^{n}\alpha_{j}{\tilde{IIT}}_{t-j}\right\}\\ & & \;\tilde{G}\left({\tilde{S}}_{it},\tilde{v},\tilde{c}\right)=\frac{1}{1+\text{exp}\left(\tilde{v}\;\left({\tilde{S}}_{it}-\tilde{c}\right)\right)} \end{array}$$

where *i* = 1, 2, … , N, where *N* = 3 and N is number of width bands, *t* = 1, 2, … , T, where T is the last year studied, namely 2017, *j* = 1, 2, … , n, where *n* = 20, and n is the range of the study period (1997–2017),where Gini coefficient $\tilde{Gini}$*,* the effect of foreign trade as the degree of economic openness (OPEN),$\;(Gini_{t-1})$ Gini coefficient of the previous period, foreign direct investment (FDI, integration of international trade (IIT), transition variable $\tilde{S}$, transition point $\tilde{c}$, transition velocity $\tilde{v}$ all of which are fuzzified. Note that ~ means the notion used is fuzzified.

## Applying the modeling cycle of FRBS in the framework of LSTAR models for estimating gini coefficient bands

**Step 0 - Stable variables** in Table 3, the unit root test shows that the null hypothesis can only be rejected after the first order differencing (1) for all the selected variables at one and 1 per cent level of significance. This is evidenced by ADF test result at the ordinary level, which shows that the computed negative ADF test statistics for each variable, is less than the Mackinnon (1991) critical value in the absolute term. Thus, the null hypothesis is accepted at level series indicating that all the variables are non-stationary at level but only became stationary after the first order unit root differencing (screening).

**Table 3:** Summary of the unit root test result.

Variables	At Level		First Order Difference	
	ADF Test Stat	Order of Integration	ADF Test Stat	Order of Integration
Gini	−2.612	–	−4.667	(1)
FDI	−2.594	–	−3.756	(1)
IIT	−1.387	–	−3.942	(1)
OPEN	−2.294	–	−4.651	(1)
Note:	Critical Value: 1% = −3.737 5% = −2.991 10% = −2.634		Critical Value: 1% = −3.737 5% = −2.991 10% = −2.635	

*Note:* Estimations for the unit root test result

* = 10% level of significance

** = 5% level of significance

*** = 1% level of significance.

**Step 1** - The LM test results for linear and nonlinear models are shown in Table 4, where the values of *K = 1, K = 2, K = 3* represent the first-, second-, and third-order Taylor's approximations, respectively.

**Table 4:** Selecting transition variables using Taylor's approximation.

Variables	Values		
	*K* *=* *1*	*K* *=* *2*	*K* *=* *3*
IIT	3.31 (0.039)	3.65 (0.044)	2.97 (0.038)
OPEN	3.99 (0.017)	4.74 (0.009)	5.44 (0.005)
FDI	3.97 (0.017)	3.64 (0.037)	3.41 (0.042)
GINI _t-1_	3.51 (0.031)	3.37 (0.025)	3.42 (0.041)

*Note:* The LM test results for selecting linear or non-linear models and transfer variable.

**Step 2** - According to Table 4, the statistical value for OPEN is higher than the critical value of other variables. So, it is selected as the transfer variable. Transition means that it has the most nonlinear behavior compared to other variables.

**Step 3** - According to the description of Step 3 of the modeling cycle in Sub-section 5.2, the LSTAR transition function is selected, as shown in Table 5.

**Table 5:** Selecting the transition function.

Test statistics	*F*_3_	*F*_2_	*F*_1_	Proposed model
Statistics LM (prob)	2.17 (0.864)	2.82 (0.632)	3.77 (0.037)	LSTAR

*Note:* P-values and F test results for selecting LSTAR transition function.

**Step 4** - The values of velocity and transfer point are estimated at 0.541 and 0.432, respectively, as shown in Table 6. See the EViews output in Appendix, Table 13, p.31.

**Table 6:** The velocity and transition points using Newton Raphson's algorithm.

Transition velocity γ	Transition point$\;c_{i}$
0.541	0.432

*Note:* Calculating transfer point c and transfer velocity point v, based on the initial approximations.

**Step 5** - The low threshold is calculated as 0.41. and the high threshold is calculated as 0.47. All the calculations of Steps 1–5 are performed in Windows 10, 64-bit and Eviews10 software.

**Step 6** - We used fuzzy logic to calculate the transition function values, as in Table 2, in three classes as high, middle, and low bounds. Accordingly, when the transition function is in the high bound, the value of the Gini coefficient is in the high bound (high width). Similarly, when the transition function is in the middle bound, the Gini coefficient is in the middle bound (front). When the transition function is in the low bound, the Gini coefficient is in the low bound (low width).

Transition variable (OPEN) and nonlinear tests are chosen according to [9,^19^,26]. The innovation of this study is in using the logistic smooth transition autoregressive model in the form of fuzzy-based rules and fuzzy database. Accordingly, the logistic smooth transition autoregressive model that is generalized by [18,^19^,27] will be as in (31). To study the calculations on fuzzy numbers refer to [28].

(31) $$\begin{array}{c} {\tilde{Gini}}_{i,t}\left(\;{\tilde{Gini}}_{t-j}\tilde{,FDI},\tilde{OPEN},\tilde{IIT,}{\tilde{S}}_{it},\tilde{c,}\tilde{v}\;\right)=\sum_{j=1}^{n}\alpha_{j}{\tilde{Gini}}_{t-j}+\sum_{j=1}^{n}\alpha_{j}{\tilde{FDI}}_{t-j}+\sum_{j=1}^{n}\alpha_{j}{\tilde{OPEN}}_{t-j}+\sum_{j=1}^{n}\alpha_{j}{\tilde{IIT}}_{t-j}\\ +\tilde{G}\left({\tilde{S}}_{it},\tilde{v},\tilde{c}\right)*\left\{\;\sum_{j=1}^{n}\alpha_{j}{\tilde{Gini}}_{t-j}+\sum_{j=1}^{n}\alpha_{j}{\tilde{FDI}}_{t-j}+\sum_{j=1}^{n}\alpha_{j}{\tilde{OPEN}}_{t-j}+\sum_{j=1}^{n}\alpha_{j}{\tilde{IIT}}_{t-j}\right\}\\ \tilde{G}\left({\tilde{S}}_{it},\tilde{v},\tilde{c}\right)=\frac{1}{1+\text{exp}\left(\tilde{v}\;\left({\tilde{S}}_{it}-\tilde{c}\right)\right)}, \end{array}$$

**Fig. 6:**
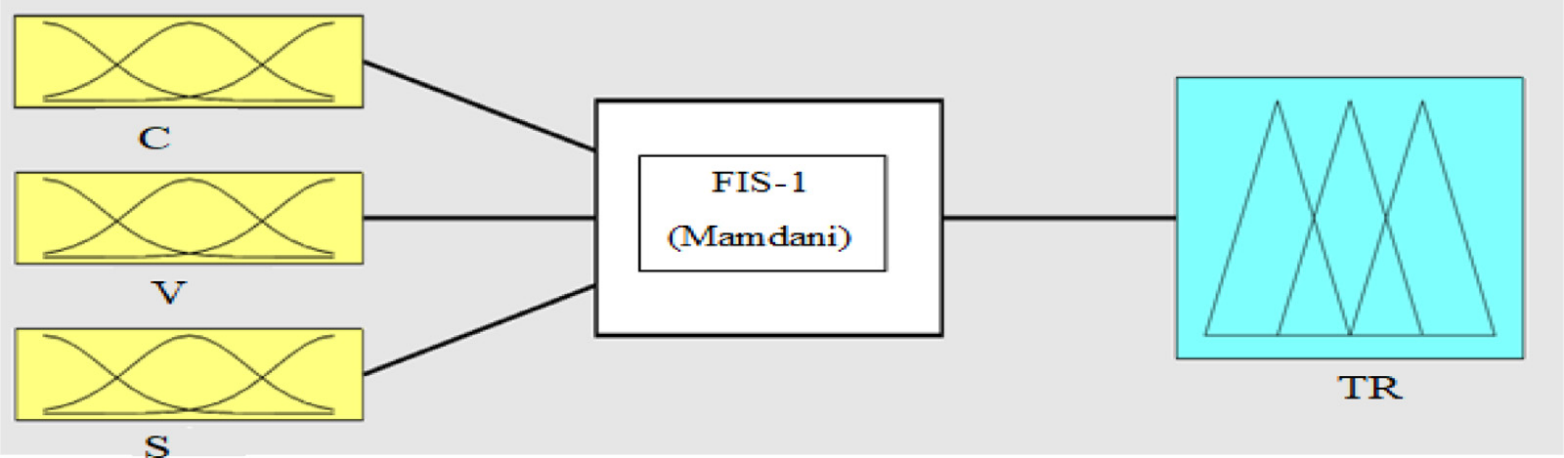
Transition function parameters (input) and transition function (output) using the fuzzy database.in Table 3.

In model (31), *i* = 1, 2, … , N, where *N* = 3 and N is number of width bands, *t* = 1, 2, … , T, where T is the last year studied, namely 2017, *j* = 1, 2, … , n, where *n* = 20, and n is the range of the study period (1997–2017), where ${\tilde{Gini}}_{i,t}\;,{\tilde{Gini}}_{t-j},\;\tilde{FDI},\tilde{OPE},\tilde{IIT,}{\tilde{S}}_{it},\tilde{c,}\tilde{v}$, respectively, refers to the Gini coefficient, the Gini coefficient of the previous period, foreign direct investment, degree of economic openness, integration of international trade, transition variable, transition point,and transition velocity, all of which are fuzzified. In this study, $\tilde{OPEN}\;$ is the transition variable, and $\tilde{G}$ is the transition function (TR). In order to obtain the transaction function in high, low, and middle bounds, in accordance with (31), the inputs are initially analyzed based on fuzzy- rules as shown in Fig. 6.

The transfer function consists of three parameters as $\tilde{c}$, $\tilde{v}$, and ${\tilde{S}}_{it}$. The value of the transfer function is calculated using the fuzzy logic and according to Table 7.

**Table 7:** Calculating transition function based on input parameters.

Bounds	Transition function parameters (input)			Transition function (output)
	Transition point c	Transition velocity v	Transition variable S (OPEN)	
Low bound	(0, 0.25)	(0, 0.21)	(0, 0. 38)	(0, 0.339)
Middle bound	(0.25, 0.375)	(0.21, 0.5)	(0. 38, 0. 67)	(0.339, 0.5)
High bound	(0.375, 0.75)	(0.5, 0.99)	(0. 67, 1)	(0.5, 1)

*Note:* Computing the value of transition function in three classes as high, middle, and low bounds based on parameters as $\tilde{c}$, $\tilde{v}$, and ${\tilde{S}}_{it}$.

**Fig. 7:**
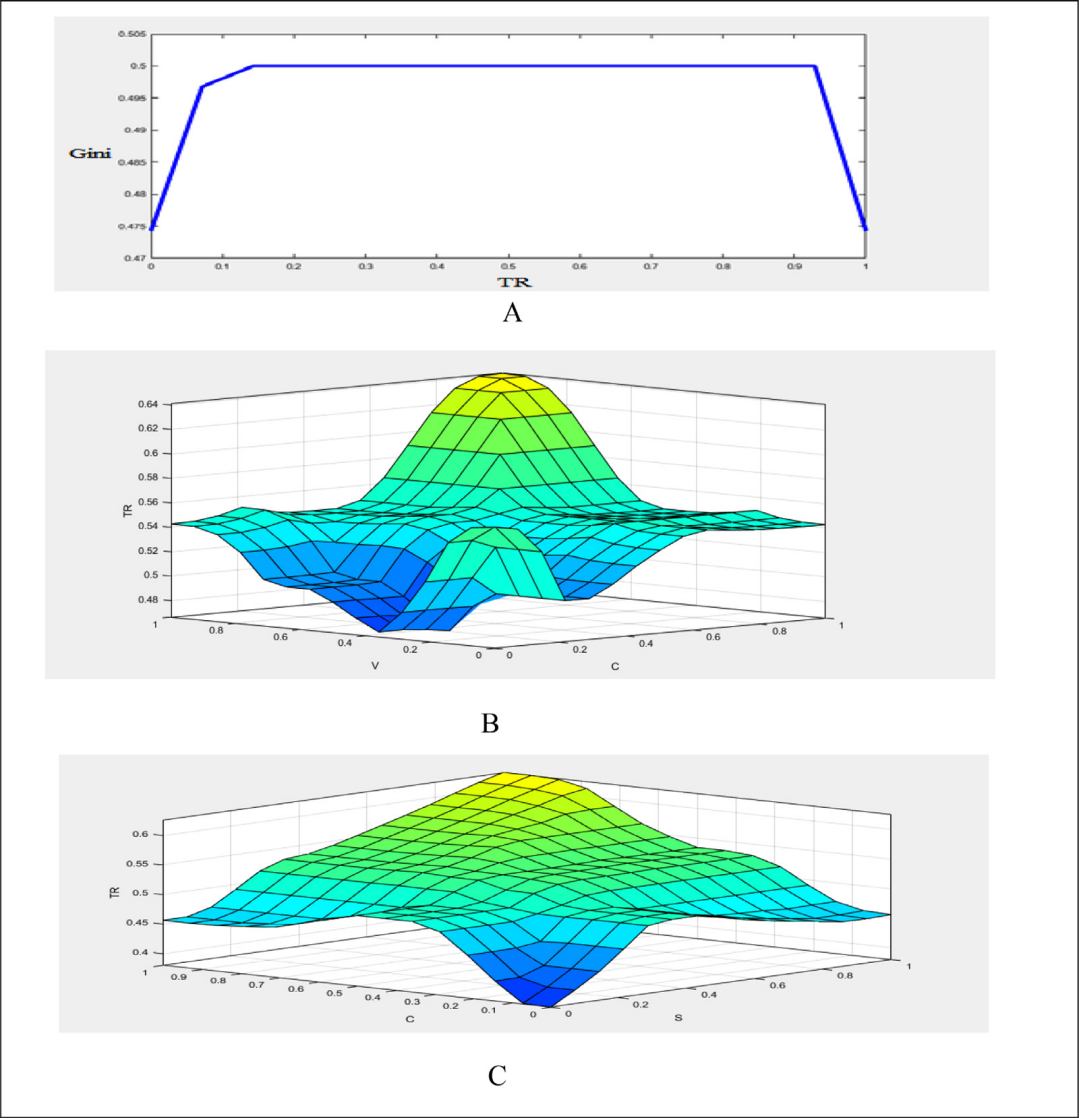
A: Transition function in terms of the transition variable (degree of economic openness, B: Transition function in terms of the transition velocity and transition point and C: Transition function in terms of the transition variable and transition point.

The values of transition function in three classes as high, middle, and low bounds are estimated. Accordingly, when the transition function is in the high bound, the value of the Gini coefficient is in the high bound (high width). Similarly, when the transition function is in the middle bound, the Gini coefficient is in the middle bound (front), and when the transition function is in the low bound, the value of Gini coefficient is in the low bound (low width). The Gaussian function is used for the membership function related to the output variable of the transition function; The Gaussian function is used because the covered domain can be carefully adjusted. It should be noted that in the present study, there are 3 fuzzy sets (low, middle, and high) and the number of input variables is 3 which are $\tilde{c}$, $\tilde{v}$, and ${\tilde{S}}_{it}$. Therefore, the number of the required rules will be 27 [29]. Fig. 7-A shows the transition function (degree of economic openness is the transition variable). The effect of the other parameters of the transition function along with the transition variable on the transition function is shown in Fig. 7-B,C.

After determining the bounds of the transition function, in the next stage, a domain is determined on each of the input variables (foreign direct investment, degree of economic openness, and integration of international trade). For this purpose, all the data are initially transferred to the values in [0,1]. Prior to drawing the membership functions, in order to specify the range of linguistic variables (foreign direct investment, degree of economic openness, and integration of international trade), the mean values, mean difference from standard deviation, and total mean and standard deviation of each variable must be calculated. In this case, the range of low linguistic variable will be from the mean to 0. The range of middle linguistic variable will be from total standard deviation and mean to the difference of standard deviation and mean. Finally, the range of high linguistic variable will be from the mean to 1. In Table 8, the values are presented for the three variables.

**Table 8:** Descriptive statistical indicators for input variables based on the calculation by the authors.

Descriptive statistical indicators	Foreign Direct Investment (FDI)	Integration of International Trade (IIT)	Degree of economic Openness (OPEN)	Gini coefficient with one the of lag (Gini t-1)
Mean	0.523	0.731	0.038	0.398
Standard deviation	0.354	0.138	0.028	0.010
Total mean and standard deviation	0.877	0.869	0.067	0.387
Difference of standard deviation and mean	0.168	0.592	0.009	0.408

*Note:* Determined domain for each of the input variables included: foreign direct investment, degree of economic openness, integration of international trade for high, middle, and low bounds.

**Fig. 8:**
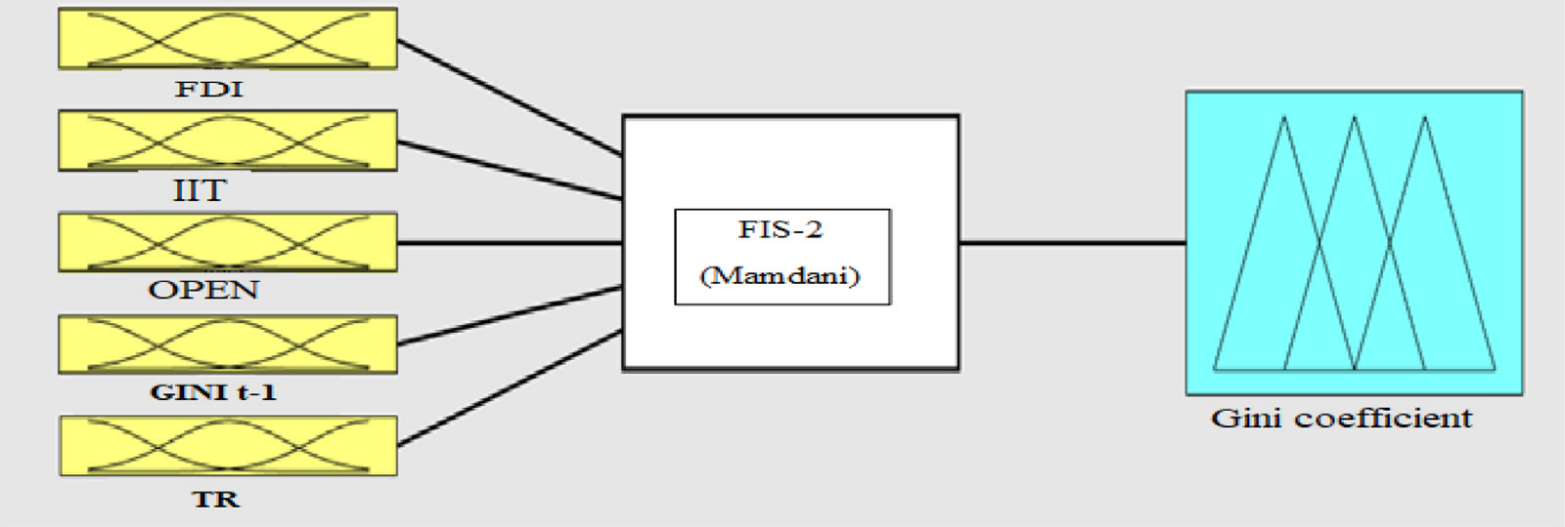
Transition function, independent variables (input) and Gini coefficient (output) using fuzzy database.

In the fuzzy inference stage, the required linguistic rules must be determined to link the input and output variables. In this study, following previous studies such as [29], to fuzzify of the above (Table 8) variables, in the first stage, the low (L), middle (M), and high (H) linguistic expressions are used for each of the input and output variables. Then, the Gaussian membership function is used for each of the linguistic expressions in each of the input variables [6,29]. Now, based on fuzzy logic and in accordance with Fig. 8, the effectiveness of transition function and independent variables for the Gini coefficient are specified. To calculate the value of the high, low, and middle bounds (proportional to the high, low, and front width) for the Gini coefficient, in accordance with Table 14 three situations (presented in Appendix) are considered for the transition function (the number of the required rules will be 243). Table 14 is obtained based on [30]. Fig. 9-A shows the effect of the transition function and transition variable (OPEN) on the Gini coefficient. The effect of other independent variables such as foreign direct investment, integration of international trade, and transition function on the Gini coefficient is shown in Fig. 9-B and C (presented in Appendix). The three-dimensional space is better for examining the effect of the two variables. In Fig. 7-A, by increasing TR and OPEN, the Gini coefficient first increases, and, again, by increasing TR and OPEN, the effect becomes constant. Finally, it decreases the Gini coefficient. In Fig. 7-B, by increasing IIT and OPEN, the Gini coefficient first increases, again by increasing IIT and OPEN, the effect becomes constant. Finally, it decreases. In Fig. 7-C, by increasing FDI and OPEN, the Gini coefficient first increases, again by increasing FDI and OPEN, the effect becomes constant and finally decreases the Gini coefficient.

**Fig. 9a:**
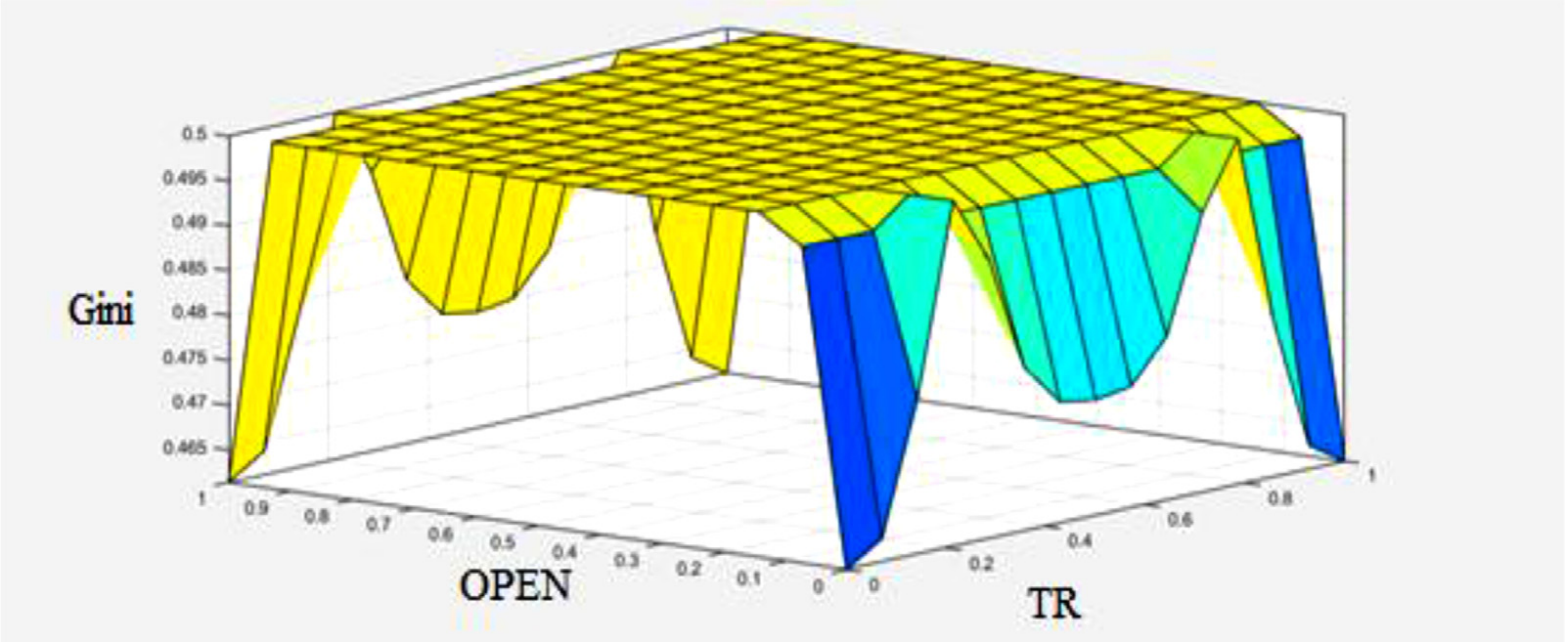
A: Effect of transition variable (OPEN) and transition function on the Gini coefficient using Eq. (31).

**Fig. 9bc:**
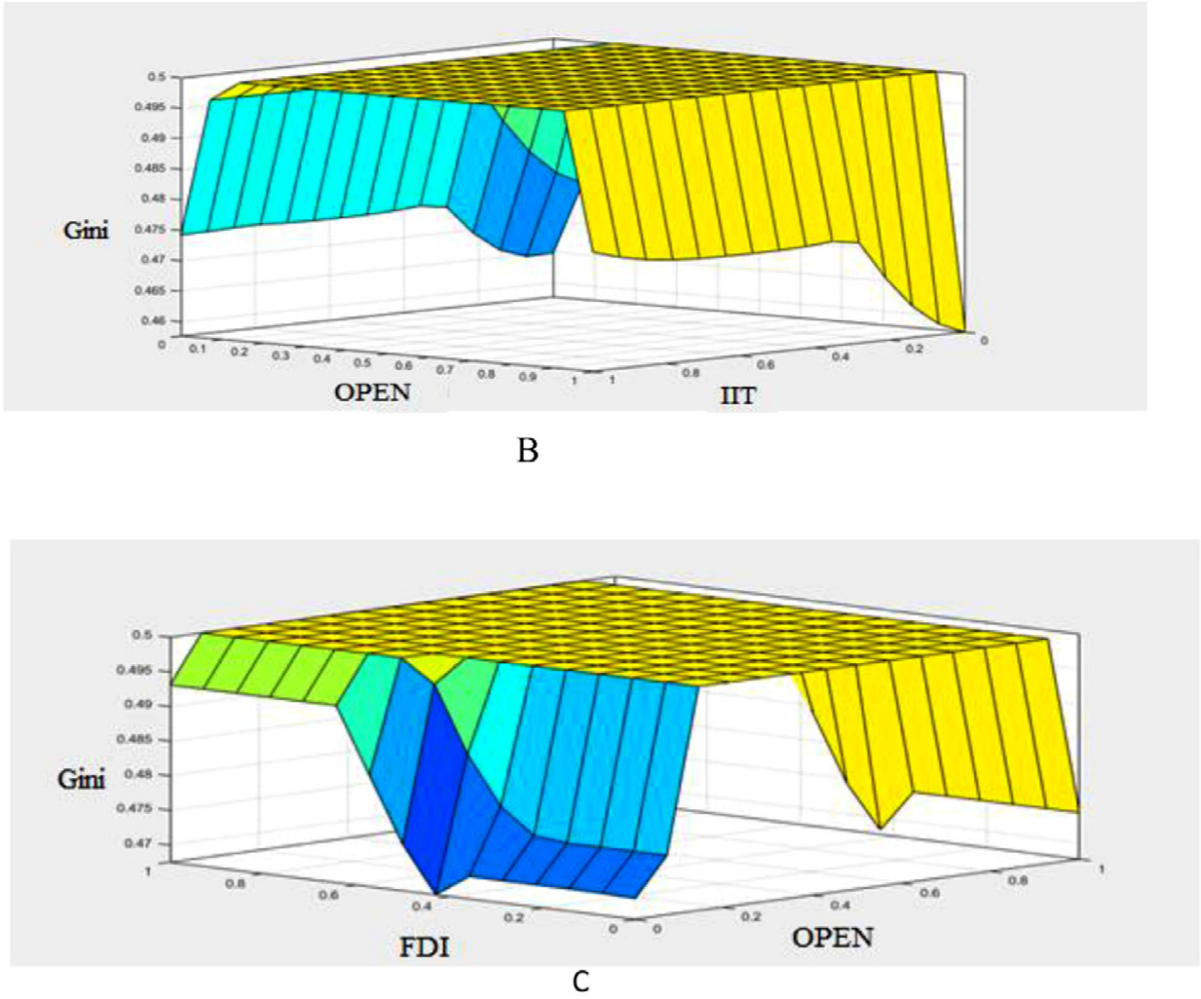
B: Effect of transition variable (OPEN) and integration of international trade on the Gini coefficient by using Eq. (31) and C: Effect of transition variable (OPEN) and foreign direct investment on the Gini coefficient by using Eq. (31).

**Step 7** - In the next stage, which is the defuzzification stage, the value of Gini coefficient is obtained. In this study, the linguistic rules are used and the linguistic dataset [6,30] is shown in Table 14. All the relevant calculations are performed on Windows 10, 64-bit and MATLAB R2018a [12].

**Fig. 10:**
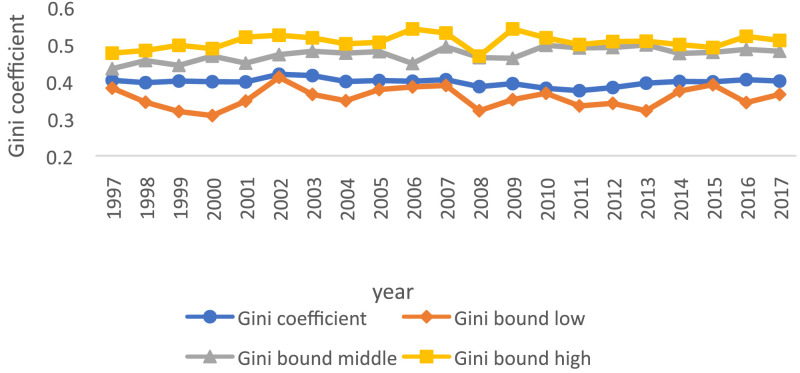
Comparing traditional value of Gini coefficient with high, low, and middle width by using Eq. (31).

These results are presented in Table 9. The high, low, and middle width of the Gini coefficient is calculated and compared with the traditional value of the Gini coefficient appeared in Fig. 10.

**Table 9:** Traditional value of Gini coefficient of high, low, and middle calculated by the author using Eq. (31).

Year	Gini coefficient	Gini bound low	Gini bound middle	Gini bound high
1997	0.402	0.382	0.434	0.476
1998	0.396	0.344	0.456	0.483
1999	0.400	0.319	0.443	0.497
2000	0.399	0.308	0.468	0.488
2001	0.398	0.347	0.449	0.519
2002	0.419	0.411	0.472	0.524
2003	0.415	0.365	0.481	0.517
2004	0.399	0.348	0.476	0.501
2005	0.402	0.378	0.48	0.505
2006	0.400	0.385	0.448	0.541
2007	0.404	0.389	0.493	0.53
2008	0.385	0.321	0.464	0.468
2009	0.393	0.351	0.462	0.541
2010	0.381	0.368	0.497	0.517
2011	0.375	0.334	0.49	0.499
2012	0.383	0.341	0.491	0.507
2013	0.395	0.321	0.498	0.508
2014	0.399	0.374	0.475	0.499
2015	0.398	0.391	0.478	0.491
2016	0.404	0.343	0.486	0.521
2017	0.400	0.365	0.481	0.51

*Note:* Comparing traditional value of Gini coefficient with the high, low, and middle width of the Gini coefficient.

## Conclusion

In this paper, a method is presented for estimating the bands of economic variables under uncertainty conditions. A practical example is given for estimating Gini coefficient of high, middle and low bands. For this purpose, the fuzzy logistic smooth transition autoregressive (FLSTAR) mode, is used which presents the application of fuzzy sets in regression. One of the merits of FLSTAR model is flexibility in modeling and strong explanatory power, because by calculating the bands of variables in economics, for example, the Gini coefficient, comparing it with the traditional value of Gini coefficient in different years, and calculating the difference between this value and other bounds (high, low, and middle), the adverse effect of long-term class gap can be specifically considered. These results are important as, they can be a guide for policy makers, because the value of the Gini coefficient can be reduced until the low width, and its current trend is not compatible with the optimal use of facilities. Therefore, in the foreign policies, it is suggested that the state pursues the policies of removing barriers to free trade and membership in World Trade Organization. The proposed approach is just one application of FLSTAR and it can be applied in the subjects such as insurance, taxes, tariffs, subsidies, and commerce.

## Declaration of Competing Interest

The authors declare that they have no known competing for financial interests or personal relationships that could have appeared to influence the work reported in this paper.

## Appendix Supplementary materials

Image, application 1

## References

[1] www.cbi.ir.

[2] Lim G.C., McNelis PD. (2014). Income Inequality, Trade and Financial Openness. Proceedings of the joint RES-SPR Conference on Macroeconomic Challenges Facing Low-Income Countries.

[3] Aznarte J.L., Medeiros MC., Benítez JM. (2010). Linearity testing for fuzzy rule-based models. Fuzzy Sets Syst..

[4] Young W.L. (1977). The Box-Jenkins approach to time series analysis and forecasting: principles and applications. RAIRO-Operations Research-Recherche Opérationnelle.

[5] H. Tong, K.S. Lim, Threshold autoregression, limit cycles and cyclical data. In: *Exploration Of A Nonlinear World: An Appreciation of Howell Tong's Contributions to Statistics*. 2009. p. 9–56.‏

[6] Teräsvirta T. (1994). Specification, estimation, and evaluation of smooth transition autoregressive models. J. Am. Stat. Assoc..

[7] Zadeh LA. (1965). Fuzzy sets. Inf. Control.

[8] Lindström T. (1998). A fuzzy design of the willingness to invest in Sweden. J. Econ. Behav. Organ..

[9] Villavicencio A.L. (2008). Nonlinearities or outliers in real exchange rates?. Econ. Model..

[10] Pourjavad E., Shahin A. (2018). The application of Mamdani fuzzy inference system in evaluating green supply chain management performance. Int. J. Fuzzy Syst..

[11] Aznarte J.L. (2008). Modelling Time Series Through Fuzzy Rule-Based models: a Statistical Approach. https://www.digibug.ugr.es/bitstream/handle/10481/2061/17674591.pdf.

[12] www.mathwork.com.

[13] Ganjoei R.A. (2020). Estimation of upper and lower bounds of Gini coefficient by fuzzy data. Data Brief.

[14] Dubois D., Prade H. (1980). Systems of linear fuzzy constraints. Fuzzy Sets Syst..

[15] Aznarte J.L. (2011). Fuzzy autoregressive rules: towards linguistic time series modeling. Econom. Rev..

[16] www.eviews.com.

[17] Luukkonen R., Saikkonen P., Teräsvirta T. (1988). Testing linearity against smooth transition autoregressive models. Biometrika.

[18] Teräsvirta T. (1998). Modelling economic relationships with smooth transition regressions. Handbook of Applied Economic Statistics.

[19] Tsay RS. (1989). Testing and modeling threshold autoregressive processes. J. Am. Stat. Assoc..

[20] Dijk D., Teräsvirta T., Franses. P.H. (2002). Smooth transition autoregressive models—A survey of recent developments. Econom. Rev..

[21] Enders W. (2008). Applied Econometric Time Series.

[22] Bonga-Bonga L. (2009). Forward exchange rate puzzle: joining the missing pieces in the Rand-US Dollar exchange market. Stud. Econ. Econom..

[23] Teräsvirta T., Lutkepohl H., Kratzig M. (2009). Smooth transition regression modeling. *Applied time series econometrics*.

[24] Teräsvirta T., Anderson HM. (1992). Characterizing nonlinearities in business cycles using smooth transition autoregressive models. J. Appl. Econom..

[25] Skalin J., Teräsvirta T. (2002). Modeling asymmetries and moving equilibria in unemployment rates. Macroecon. Dyn..

[26] Milanovic B. (2010). Can We Discern the Effect of Globalization on Income Distribution? Evidence from Household Surveys. World Bank Econ. Rev..

[27] Hesamian G., Akbari M.G. (2017). Semi-parametric partially logistic regression model with exact inputs and intuitionistic fuzzy outputs. Appl. Soft. Comput..

[28] Mareš M. (1997). Weak arithmetics of fuzzy numbers. Fuzzy Sets Syst..

[29] Draeseke R., Giles D.E.A. (2002). A fuzzy logic approach to modelling the New Zealand underground economy. Math. Comput. Simul..

[30] Lee C-C. (1990). Fuzzy logic in control systems: fuzzy logic controller. I. IEEE Trans. Syst. Man Cybern..

