# Supplementary material for: A method for estimating width bands of variables in economics under uncertainty conditions
Source: MethodsX. 2020 Dec 14;8:101184. doi: 10.1016/j.mex.2020.101184 (PMC7753204; doi:10.1016/j.mex.2020.101184)
Supplement: Supplementary file 1 [file mmc1.docx]

**Appendix**

Table 1: Results of Taylor's first approximation for the foreign direct investment variable

| **Variables Coefficient Std. Error T-statistic Prob**  *Constant*  0.006 0.065 3.359 0.048  ${Gini}_{t-1}$ 0.008 0.048 8.652 0.000  ${FDI}_{t-1}$ 0.023 0.165 5.023 0.021  ${OPEN}_{t-1}$ - 0.620 0.003 - 4.265 0.811  ${IIT}_{t-1}$ 0.039 0.069 2.956 0.062  ${FDI}_{t-1}*{Gini}_{t-1}$ 0.045 0.236 3.980 0.035  ${FDI}_{t-1}^{2}$ - 0.003 0.300 - 4.036 0.021  ${FDI}_{t-1}*{OPEN}_{t-1}$ 0.032 0.754 3.897 0.034  ${FDI}_{t-1}*{IIT}_{t-1}$ - 0.064 0.003 - 7.811 0.000  $\varepsilon_{t,}$ 0.003 0.055 4.632 0.034 |
| --- |
|  |
| R-squared 0.821 Mean dependent var 0.398  Adjusted R-squared 0.614 S.D. dependent var 0.010  S.E. of regression 0.002 Akaike info criterion -4.365  Sum squared resid 3.681 Schwarz criterion -5.532  Log likelihood 215.104 Hannan-Quinn criter. -4.231  F-statistic 4.654 Durbin-Watson stat 1.953  Prob(F-statistic) 0.032 |

Source: E-View Econometric Computer Software Application.

Table 2: Results of Taylor's second approximation for the foreign direct investment variable

| **Variables Coefficient Std. Error T-statistic Prob**  **Constant** 0.005 0.053 4.435 0.035  ${Gini}_{t-1}$ 0.035 0.069 4.237 0.032  ${FDI}_{t-1}$ 0.053 0.236 3.457 0.042  ${OPEN}_{t-1}$ - 0.356 0.098 - 5.452 0.010  ${IIT}_{t-1}$ 0.005 0.088 3.265 0.056  ${FDI}_{t-1}*{Gini}_{t-1}$ - 0.065 0.051 - 4.257 0.035  ${FDI}_{t-1}^{2}$ 0.036 0.099 1.567 0.652  ${FDI}_{t-1}*{OPEN}_{t-1}$ - 0.066 0.011 -3.125 0.053  ${FDI}_{t-1}*{IIT}_{t-1}$ 0.369 0.061 3.984 0.042  ${Gini}_{t-1}*{FDI}_{t-1}^{2}$ 0.004 0.059 5.586 0.012  ${FDI}_{t-1}^{3}$ 0.069 0.023 3.567 0.051  ${OPEN}_{t-1}*{FDI}_{t-1}^{2}$ - 0.326 0.203 - 4.568 0.034  ${IIT}_{t-1}*{FDI}_{t-1}^{2}$ - 0.001 0.022 -5.120 0.021  $\varepsilon_{t,}$ 0.098 0.056 3.852 0.045 |
| --- |
|  |
| R-squared 0.795 Mean dependent var 0.398  Adjusted R-squared 0.652 S.D. dependent var 0.010  S.E. of regression 0.005 Akaike info criterion -4.373  Sum squared resid 4.236 Schwarz criterion -4.778  Log likelihood 115.104 Hannan-Quinn criter. -5.326  F-statistic 4.230 Durbin-Watson stat 1.895  Prob (F-statistic) 0.023 |

Source: E-View Econometric Computer Software Application.

Table 3: Results of Taylor's third approximation for the foreign direct investment variable

| **Variables Coefficient Std. Error T-statistic Prob**  **Constant** 0.006 0.045 4.023 0.034  ${Gini}_{t-1}$ 0.012 0.023 5.321 0.002  ${FDI}_{t-1}$ - 0.156 0.036 - 3.923 0.042  ${OPEN}_{t-1}$ 0.022 0.078 7.235 0.008  ${IIT}_{t-1}$ - 0.324 0.032 - 6.023 0.005  ${FDI}_{t-1}*{Gini}_{t-1}$ 0.542 0.055 3.894 0.041  ${FDI}_{t-1}^{2}$ 0.002 0.036 4.236 0.031  ${FDI}_{t-1}*{OPEN}_{t-1}$ - 0.059 0.044 - 5.236 0.013  ${FDI}_{t-1}*{IIT}_{t-1}$ 0.066 0.230 3.875 0.045  ${Gini}_{t-1}*{FDI}_{t-1}^{2}$ - 0.006 0.089 - 3.952 0.045  ${FDI}_{t-1}^{3}$ 0.055 0.026 4.023 0.032  ${OPEN}_{t-1}*{FDI}_{t-1}^{2}$ - 0.896 0.003 - 6.236 0.005  ${IIT}_{t-1}*{FDI}_{t-1}^{2}$ 0.452 0.022 4.236 0.032  ${Gini}_{t-1}*{FDI}_{t-1}^{3}$ 0.633 0.052 3.125 0.042  ${FDI}_{t-1}^{4}$ 0.733 0.002 1.235 0.564  ${FDI}_{t-1}^{3}*{OPEN}_{t-1}$ - 0.044 0.235 - 2.123 0.452  ${FDI}_{t-1}^{3}*{IIT}_{t-1}$ 0.336 0.098 5.236 0.002  $\varepsilon_{t,}$ 0.056 0.045 3.925 0.045 |
| --- |
|  |
| R-squared 0.854 Mean dependent var 0.398  Adjusted R-squared 0.782 S.D. dependent var 0.010  S.E. of regression 0.005 Akaike info criterio -3.256  Sum squared resid 4.001 Schwarz criterion -4.568  Log likelihood 169.326 Hannan-Quinn criter. -4.230  F-statistic 5.920 Durbin-Watson stat 2.023  Prob (F-statistic) 0.009 |

Source: E-View Econometric Computer Software Application.

Table 4: Results of Taylor's first approximation for the degree of economic openness variable

| **Variables Coefficient Std. Error T-statistic Prob**  *Constant*  0.032 0.023 6.023 0.002  ${Gini}_{t-1}$ 0.036 0.126 4.236 0.032  ${FDI}_{t-1}$ - 0.087 0.032 -3.256 0.051  ${OPEN}_{t-1}$ 0.456 0.001 4.230 0.032  ${IIT}_{t-1}$ - 0.008 0.032 -3.265 0.053  ${OPEN}_{t-1}*{Gini}_{t-1}$ -0.213 0.005 6.235 0.002  ${OPEN}_{t-1}*{FDI}_{t-1}$ 0.018 0.023 3.980 0.031  ${OPEN}_{t-1}^{2}$ 0.235 0.810 4.278 0.032  ${OPEN}_{t-1}*{IIT}_{t-1}$ -0.542 0.001 -2.501 0.062  $\varepsilon_{t,}$ 0.056 0.056 4.102 0.042 |
| --- |
|  |
| R-squared 0.781 Mean dependent va 0.398  Adjusted R-squared 0.645 S.D. dependent var 0.010  S.E. of regression 0.006 Akaike info criterion -3.373  Sum squared resid 4.203 Schwarz criterion -5.778  Log likelihood 123.023 Hannan-Quinn criter. -4.232  F-statistic 5.893 Durbin-Watson stat 2.110  Prob(F-statistic) 0.003 |

Source: E-View Econometric Computer Software Application.

Table 5: Results of Taylor's second approximation for the degree of economic openness variable

| **Variables Coefficient Std. Error T-statistic Prob**  **Constant** 0.056 0.021 3.032 0.061  ${Gini}_{t-1}$ 0.095 0.078 5.098 0.012  ${FDI}_{t-1}$ - 0.630 0.002 - 2.058 0.563  ${OPEN}_{t-1}$ 0.005 0.078 1.954 0.811  ${IIT}_{t-1}$ - 0.648 0.065 - 6.235 0.003  ${OPEN}_{t-1}*{Gini}_{t-1}$ - 0.035 0.102 - 4.845 0.026  ${OPEN}_{t-1}*{FDI}_{t-1}$ 0.784 0.004 4.542 0.039  ${OPEN}_{t-1}^{2}$ - 0.300 0.032 - 5.032 0.001  ${OPEN}_{t-1}*{IIT}_{t-1}$ 0.014 0.042 3.945 0.042  ${Gini}_{t-1}*{OPEN}_{t-1}^{2}$ -0.002 0.064 - 4.065 0.028  ${FDI}_{t-1}*{OPEN}_{t-1}^{2}$ 0.588 0.070 4.542 0.024  ${OPEN}_{t-1}^{3}$ 0.006 0.089 1.802 0.542  ${IIT}_{t-1}*{OPEN}_{t-1}^{2}$ - 0.008 0.068 - 1.680 0.421  $\varepsilon_{t,}$ 0.006 0.082 5.023 0.012 |
| --- |
|  |
| R-squared 0.862 Mean dependent var 0.398  Adjusted R-squared 0.721 S.D. dependent var 0.010  S.E. of regression 0.006 Akaike info criterion -5.326  Sum squared resid 3.851 Schwarz criterion -4.326  Log likelihood 132.548 Hannan-Quinn criter. -3.894  F-statistic 7.920 Durbin-Watson stat 2.007  Prob(F-statistic) 0.003 |

Source: E-View Econometric Computer Software Application.

| **Variables Coefficient Std. Error T-statistic Prob**  **Constant** 0.256 0.005 3.892 0.035  ${Gini}_{t-1}$ 0.500 0.033 5.023 0.005  ${FDI}_{t-1}$ 0.950 0.088 1.811 0.154  ${OPEN}_{t-1}$ - 0.680 0.022 - 6.280 0.002  ${IIT}_{t-1}$ 0.201 0.007 4.400 0.032  ${OPEN}_{t-1}*{Gini}_{t-1}$ - 0.005 0.011 - 4.606 0.032  ${OPEN}_{t-1}*{FDI}_{t-1}$ 0.068 0.002 3.820 0.048  ${OPEN}_{t-1}^{2}$ - 0.810 0.094 - 5.002 0.032  ${OPEN}_{t-1}*{IIT}_{t-1}$ 0.000 0.044 2.899 0.238  ${Gini}_{t-1}*{OPEN}_{t-1}^{2}$ - 0.652 0.061 - 2.904 0.539  $\left( {FDI}_{t-1}*{OPEN}_{t-1}^{2} \right)$ 0.056 0.022 3.912 0.042  ${OPEN}_{t-1}^{3}$ 0.950 0.056 7.200 0.002  ${IIT}_{t-1}*{OPEN}_{t-1}^{2}$ - 0.006 0.011 - 5.811 0.032  ${Gini}_{t-1}*{OPEN}_{t-1}^{3}$ 0.300 0.022 3.621 0.049  ${FDI}_{t-1}*{OPEN}_{t-1}^{3}$ - 0.320 0.050 - 3.602 0.048  ${OPEN}_{t-1}^{4}$ - 0.811 0.080 - 4.500 0.023  ${IIT}_{t-1}*{OPEN}_{t-1}^{3}$ 0.720 0.093 3.505 0.042  $\varepsilon_{t,}$ 0.030 0.002 4.702 0.049 |
| --- |
|  |
| R-squared 0.811 Mean dependent var 0.398  Adjusted R-squared 0.604 S.D. dependent var 0.010  S.E. of regression 0.001 Akaike info criterion -9.373  Sum squared resid 3.650 Schwarz criterion -8.778  Log likelihood 115.104 Hannan-Quinn criter. -9.232  F-statistic 6.920 Durbin-Watson stat 2.1667  Prob(F-statistic) 0.008 |

Table 6: Results of Taylor's third approximation for the degree of economic openness variable

Source: E-View Econometric Computer Software Application.

Table 7: Results of Taylor's first approximation for the integration of international trade variable

| **Variables Coefficient Std. Error T-statistic Prob**  *Constant*  0.001 0.087 2.542 0.081  ${Gini}_{t-1}$ 0.085 0.021 3.359 0.002  ${FDI}_{t-1}$ 0.051 0.231 3.325 0.000  ${OPEN}_{t-1}$ -0.257 0.800 - 5.548 0.024  ${IIT}_{t-1}$ 0.591 0.008 3.751 0.035  ${IIT}_{t-1}*{Gini}_{t-1}$ 0.000 0.048 4.824 0.001  ${IIT}_{t-1}*{FDI}_{t-1}$ -0.013 0.006 -2.826 0.478  ${IIT}_{t-1}*{OPEN}_{t-1}$ -0.011 0.001 -4.654 0.004  ${IIT}_{t-1}^{2}$ 0.015 0.003 3.915 0.025  $\varepsilon_{t,}$ 0.011 0.014 3.321 0.002 |
| --- |
|  |
| R-squared 0.811 Mean dependent var 0.398  Adjusted R-squared 0.604 S.D. dependent var 0.010  S.E. of regression 0.001 Akaike info criterion -4.973  Sum squared resid 3.662 Schwarz criterion -3.878  Log likelihood 115.104 Hannan-Quinn criter. -3.532  F-statistic 7.920 Durbin-Watson stat 2.166  Prob(F-statistic) 0.009 |

Source: E-View Econometric Computer Software Application.

Table 8: Results of Taylor's second approximation for the integration of international trade variable

| **Variables Coefficient Std. Error T-statistic Prob**  **Constant** 0.001 0.054 5.852 0.007  ${Gini}_{t-1}$ 0.023 0.025 3.364 0.021  ${FDI}_{t-1}$ - 0.302 0.236 - 6.321 0.003  ${OPEN}_{t-1}$ - 0.021 0.025 - 2.954 0.082  ${IIT}_{t-1}$ 0.032 0.021 5.811 0.023  ${IIT}_{t-1}*{Gini}_{t-1}$ 0.320 0.032 3.654 0.042  ${IIT}_{t-1}*{FDI}_{t-1}$ - 0.811 0.012 - 1.541 0.895  ${IIT}_{t-1}*{OPEN}_{t-1}$ - 0.021 0.039 - 2.823 0.062  ${IIT}_{t-1}^{2}$ 0.800 0.659 5.648 0.006  ${Gini}_{t-1}*{IIT}_{t-1}^{2}$ 0.002 0.510 7.690 0.000  ${FDI}_{t-1}*{IIT}_{t-1}^{2}$ - 0.069 0.021 - 2.569 0.521  ${OPEN}_{t-1}*{IIT}_{t-1}^{2}$ 0.320 0.365 8.361 0.000  ${IIT}_{t-1}^{3}$ 0.365 0.002 7.326 0.000  $\varepsilon_{t,}$ 0.002 0.036 5.952 0.004 |
| --- |
|  |
| R-squared 0.751 Mean dependent var 0.398  Adjusted R-squared 0.662 S.D. dependent var 0.010  S.E. of regression 0.009 Akaike info criterion -3.236  Sum squared resid 4.652 Schwarz criterion -4.694  Log likelihood 189.485 Hannan-Quinn criter. -5.623  F-statistic 6. 315 Durbin-Watson stat 2.003  Prob(F-statistic) 0.006 |

Source: E-View Econometric Computer Software Application.

| **Variables Coefficient Std. Error T-statistic Prob**  **Constant** 0.002 0.014 3.241 0.054  ${Gini}_{t-1}$ 0.210 0.015 4.487 0.021  ${FDI}_{t-1}$ - 0.321 0.784 - 2.847 0.130  ${OPEN}_{t-1}$ 0.652 0.345 3.985 0.032  ${IIT}_{t-1}$ 0.036 0.547 4.865 0.021  ${IIT}_{t-1}*{Gini}_{t-1}$ 0.954 0.368 5.641 0.001  ${IIT}_{t-1}*{FDI}_{t-1}$ - 0.036 0.695 - 5.215 0.002  ${IIT}_{t-1}*{OPEN}_{t-1}$ - 0.203 0.481 - 4.975 0.054  ${IIT}_{t-1}^{2}$ 0.369 0.120 1.541 0.874  ${Gini}_{t-1}*{IIT}_{t-1}^{2}$ 0.016 0.049 6.151 0. 005  ${FDI}_{t-1}*{IIT}_{t-1}^{2}$ - 0.003 0.850 - 2.035 0.521  ${OPEN}_{t-1}*{IIT}_{t-1}^{2}$ 0.623 0.630 1.487 0.847  ${IIT}_{t-1}^{3}$ 0.658 0.920 4.348 0.043  ${Gini}_{t-1}*{IIT}_{t-1}^{3}$ 0.325 0.482 3.841 0.041  ${FDI}_{t-1}*{IIT}_{t-1}^{3}$ - 0.564 0.036 - 3.245 0.035  ${OPEN}_{t-1}*{IIT}_{t-1}^{3}$ - 0.654 0.695 - 3.321 0.068  ${IIT}_{t-1}^{4}$ 0.032 0.298 2.865 0.068  $\varepsilon_{t,}$ 0.032 0.089 4.987 0.004 |
| --- |
|  |
| R-squared 0.689 Mean dependent var 0.398  Adjusted R-squared 0.651 S.D. dependent var 0.010  S.E. of regression 0.065 Akaike info criterion -5.369  Sum squared resid 4.235 Schwarz criterion -4.369  Log likelihood 195.235 Hannan-Quinn criter. -3.269  F-statistic 3.895 Durbin-Watson stat 2.100  Prob (F-statistic) 0.019 |

Table 9: Results of Taylor's third approximation for the integration of international trade variable

Source: E-View Econometric Computer Software Application.

Table 10: Results of Taylor's first approximation for theGini coefficient of the previous period

| **Variables Coefficient Std. Error T-statistic Prob**  *Constant*  0.005 0.000 5.048 0.001  ${Gini}_{t-1}$ 0.600 0.021 4.320 0.032  ${FDI}_{t-1}$ - 0.058 0.063 - 3.733 0.041  ${OPEN}_{t-1}$ 0.036 0.007 4.722 0.038  ${IIT}_{t-1}$ - 0.100 0.051 - 1.966 0.621  $\mathrm{Gini}_{t-1}^{2}$ 0.702 0.050 5.630 0.031  ${FDI}_{t-1}*{Gini}_{t-1}$ 0.069 0.083 3.684 0.039  ${OPEN}_{t-1}*{Gini}_{t-1}$ - 0.096 0.032 - 3.952 0.041  ${IIT}_{t-1}*{Gini}_{t-1}$ - 0.085 0.002 - 2.821 0.621  $\varepsilon_{t,}$ 0.002 0.004 4.901 0.032 |
| --- |
|  |
| R-squared 0.877 Mean dependent var 0.398  Adjusted R-squared 0.792 S.D. dependent var 0.010  S.E. of regression 0.004 Akaike info criterion -4.373  Sum squared resid 3.532 Schwarz criterion -3.778  Log likelihood 128.133 Hannan-Quinn criter. -4.232  F-statistic 5.965 Durbin-Watson stat 2.166  Prob(F-statistic) 0.009 |

Source: E-View Econometric Computer Software Application.

Table 11: Results of Taylor's second approximation for theGini coefficient of the previous period

| **Variables Coefficient Std. Error T-statistic Prob**  **Constant** 0.098 0.001 4.800 0.041  ${Gini}_{t-1}$ - 0.020 0.021 - 3.902 0.042  ${FDI}_{t-1}$ 0.050 0.069 8.025 0.000  ${OPEN}_{t-1}$ - 0.069 0.005 - 5.302 0.008  ${IIT}_{t-1}$ 0.082 0.054 5.250 0.009  $\mathrm{Gini}_{t-1}^{2}$ 0.800 0.054 6.200 0.006  ${FDI}_{t-1}*{Gini}_{t-1}$ - 0.620 0.085 - 3.883 0.042  ${OPEN}_{t-1}*\mathrm{Gini}_{t-1}$ 0.570 0.005 3.732 0.048  ${IIT}_{t-1}*\mathrm{Gini}_{t-1}$ - 0.601 0.054 - 1.953 0.762  $\mathrm{Gini}_{t-1}^{3}$ 0.602 0.009 2.817 0.411  ${FDI}_{t-1}*\mathrm{Gini}_{t-1}^{2}$ 0.097 0.083 3.910 0.042  ${OPEN}_{t-1}*\mathrm{Gini}_{t-1}^{2}$ - 0.520 0.099 - 3.703 0.041  ${IIT}_{t-1}*\mathrm{Gini}_{t-1}^{2}$ - 0.811 0.044 - 4.810 0.023  $\varepsilon_{t,}$ 0.008 0.099 4.902 0.035 |
| --- |
|  |
| R-squared 0.865 Mean dependent var 0.398  Adjusted R-squared 0.665 S.D. dependent var 0.010  S.E. of regression 0.002 Akaike info criterion -5.373  Sum squared resid 4.852 Schwarz criterion -3.778  Log likelihood 115.652 Hannan-Quinn criter -4.232  F-statistic 5.920 Durbin-Watson stat 2.166  Prob(F-statistic) 0.009 |

Source: E-View Econometric Computer Software Application.

Table 12: Results of Taylor's third approximation for theGini coefficient of the previous period

| **Variables Coefficient Std. Error T-statistic Prob**  **Constant** 0.032 0.002 3.810 0.041  ${Gini}_{t-1}$ 0.231 0.099 5.845 0.008  ${FDI}_{t-1}$ 0.210 0.064 4.865 0.031  ${OPEN}_{t-1}$ 0.065 0.003 5.803 0.005  ${IIT}_{t-1}$ - 0.321 0.085 - 5.471 0.001  $\mathrm{Gini}_{t-1}^{2}$ 0.329 0.056 3.903 0.042  ${FDI}_{t-1}*{Gini}_{t-1}$ - 0.006 0.032 - 4.120 0.031  ${OPEN}_{t-1}*\mathrm{Gini}_{t-1}$ 0.065 0.065 3.801 0.051  ${IIT}_{t-1}*\mathrm{Gini}_{t-1}$ - 0.260 0.005 - 1.942 0.421  $\mathrm{Gini}_{t-1}^{3}$ 0.478 0.005 6.004 0.003  ${FDI}_{t-1}*\mathrm{Gini}_{t-1}^{2}$ 0.850 0.065 5.104 0.021  ${OPEN}_{t-1}*\mathrm{Gini}_{t-1}^{2}$ - 0.008 0.032 - 5.019 0.041  ${IIT}_{t-1}*\mathrm{Gini}_{t-1}^{2}$ 0.056 0.005 2.910 0.214  $\mathrm{Gini}_{t-1}^{4}$ 0.000 0.014 4.301 0.031  ${FDI}_{t-1}*\mathrm{Gini}_{t-1}^{3}$ - 0.810 0.002 - 4.701 0.041  ${OPEN}_{t-1}*\mathrm{Gini}_{t-1}^{3}$ - 0.300 0.065 - 3.870 0.048  ${IIT}_{t-1}*\mathrm{Gini}_{t-1}^{3}$ 0.811 0.036 5.073 0.012  $\varepsilon_{t,}$ 0.005 0.002 5.302 0.036 |
| --- |
|  |
| R-squared 0.861 Mean dependent var 0.398  Adjusted R-squared 0.744 S.D. dependent var 0.010  S.E. of regression 0.005 Akaike info criterion -4.373  Sum squared resid 3.845 Schwarz criterion -4.778  Log likelihood 182.184 Hannan-Quinn criter. -5.232  F-statistic 6.920 Durbin-Watson stat 1.866  Prob (F-statistic) 0.059 |

Source: E-View Econometric Computer Software Application.

Table 13: Results estimation the transition velocity $v$ and the transition point$c_{i}$

| **Variables Coefficient Std. Error T-statistic Prob** |
| --- |
| (**Linear part**)  *Constant*  0.024 0.007 4.042 0.042  ${Gini}_{t-1}$ 0.198 0.050 4.892 0.036  ${FDI}_{t-1}$ 0.058 0.090 2.548 0.062  ${OPEN}_{t-1}$ - 0.080 0.044 - 4.364 0.047  ${IIT}_{t-1}$ - 0.098 0.080 - 1.912 0.721 |
| ( **Nonlinear part**)  *Constant*  0.024 0.008 3.048 0.068  ${Gini}_{t-1}$ 0.094 0.042 4.842 0.042  ${FDI}_{t-1}$ 0.010 0.047 3.733 0.045  ${OPEN}_{t-1}$ - 0.048 0.018 - 4.722 0.035  ${IIT}_{t-1}$ - 0.027 0.083 - 1.966 0.641 |
| $\varepsilon_{t,}$ 0.008 0.002 3.980 0.045  $v$ 0.541  $c$ 0.432 |
|  |
| R-square 0.881 Mean dependent va 0.398  Adjusted R-squared 0.755 S.D. dependent var 0.010  S.E. of regression 0.004 Akaike info criteri -4.452  Sum squared resid 3.692 Schwarz criterion -3.973 |

Source: E-View Econometric Computer Software Application

Table 14: Fuzzy database, (Low (L), High (H), middle (M)

| ROW | TR | IIT | GINI t-1 | FDI | OPEN | GINI | Degree | ROW | TR | IIT | GINI t-1 | FDI | OPEN | JINI | Degree | ROW | TR | IIT | GINI t-1 | FDI | OPEN | GINI | Degree |
| --- | --- | --- | --- | --- | --- | --- | --- | --- | --- | --- | --- | --- | --- | --- | --- | --- | --- | --- | --- | --- | --- | --- | --- |
| 1 | L | H | M | H | H | H | 1 | 28 | H | H | M | H | H | H | 1 | 55 | M | H | H | H | H | H | **1** |
| 2 | L | H | L | H | M | H | 1 | 29 | H | H | L | H | M | H | 1 | 56 | M | H | H | H | M | H | 1 |
| 3 | L | H | M | H | L | H | 1 | 30 | H | H | L | H | L | H | 1 | 57 | M | H | M | H | L | H | 1 |
| 4 | L | H | H | M | H | M | 1 | 31 | H | H | L | M | H | M | 1 | 58 | M | H | M | M | H | M | 1 |
| 5 | L | H | L | M | M | M | 1 | 32 | H | H | H | M | M | M | 1 | 59 | M | H | M | M | M | M | 1 |
| 6 | L | H | M | M | L | M | 1 | 33 | H | H | H | M | L | M | 1 | 60 | M | H | L | M | L | M | 1 |
| 7 | L | H | L | L | H | M | 1 | 34 | H | H | H | L | H | M | 1 | 61 | M | H | L | L | H | M | 1 |
| 8 | L | H | H | L | M | M | 1 | 35 | H | H | M | L | M | M | 1 | 62 | M | H | L | L | M | M | 1 |
| 9 | L | H | L | L | L | L | 1 | 36 | H | H | M | L | L | M | 1 | 63 | M | H | H | L | L | M | 1 |
| 10 | L | M | M | H | H | M | 1 | 37 | H | M | M | H | H | M | 1 | 64 | M | M | H | H | H | M | 1 |
| 11 | L | M | M | H | M | M | 1 | 38 | H | M | L | H | M | M | 1 | 65 | M | M | H | H | M | M | 1 |
| 12 | L | M | M | H | L | M | 1 | 39 | H | M | L | H | L | M | 1 | 66 | M | M | M | H | L | M | 1 |
| 13 | L | M | L | M | H | L | 1 | 40 | H | M | M | M | H | L | 1 | 67 | M | M | H | M | H | M | 1 |
| 14 | L | M | L | M | M | L | 1 | 41 | H | M | L | M | M | L | 1 | 68 | M | M | L | M | M | M | **1** |
| 15 | L | M | L | M | L | L | 1 | 42 | H | M | H | M | L | L | 1 | 69 | M | M | M | M | L | M | 1 |
| 16 | L | M | H | L | H | M | 1 | 43 | H | M | M | L | H | M | 1 | 70 | M | M | L | L | H | M | 1 |
| 17 | L | M | H | L | M | M | 1 | 44 | H | M | L | L | M | M | 1 | 71 | M | M | M | L | M | M | 1 |
| 18 | L | M | H | L | L | L | 1 | 45 | H | M | H | L | L | M | 1 | 72 | M | M | H | L | L | M | 1 |
| 19 | L | L | M | H | H | M | 1 | 46 | H | L | M | H | H | H | 1 | 73 | M | L | L | H | H | H | 1 |
| 20 | L | L | M | H | M | M | 1 | 47 | H | L | L | H | M | M | 1 | 74 | M | L | M | H | M | M | 1 |
| 21 | L | L | M | H | L | L | 1 | 48 | H | L | H | H | L | H | 1 | 75 | M | L | L | H | L | H | 1 |
| 22 | L | L | M | M | H | L | 1 | 49 | H | L | M | M | H | M | 1 | 76 | M | L | H | M | H | M | 1 |
| 23 | L | L | L | M | M | L | 1 | 50 | H | L | L | M | M | L | 1 | 77 | M | L | L | M | M | M | 1 |
| 24 | L | L | H | M | L | L | 1 | 51 | H | L | H | M | L | L | 1 | 78 | M | L | M | M | L | L | 1 |
| 25 | L | L | M | L | H | L | 1 | 52 | H | L | M | L | H | M | 1 | 79 | M | L | M | L | H | M | **1** |
| 26 | L | L | L | L | M | L | 1 | 53 | H | L | H | L | M | M | 1 | 80 | M | L | M | L | M | M | **1** |
| 27 | L | L | H | L | L | L | 1 | 54 | H | L | M | L | L | M | 1 | 81 | M | L | L | L | L | L | **1** |
| ... | ... | ... | ... | ... | ... | ... | ... | ... | ... | ... | ... | ... | ... | ... | ... | ... | ... | ... | ... | ... | ... | ... | **...** |
| 189 | L | H | L | M | M | H | 1 | 216 | H | L | L | M | H | L | 1 | 243 | M | L | M | L | L | L | **1** |

| 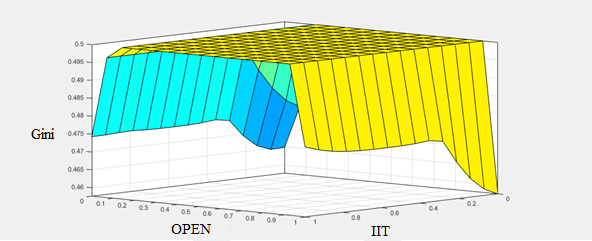  B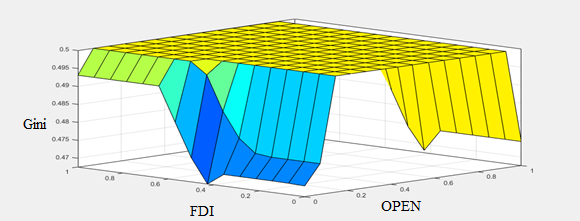  C |
| --- |

**Fig.9-B: Effect of transition variable (OPEN) and integration of international trade on the Gini coefficient by using equation (31) and C: Effect of transition variable (OPEN) and foreign direct investment on the Gini coefficient by using equation (31)**
